# A random walk model that accounts for space occupation and movements of a large herbivore

**DOI:** 10.1038/s41598-021-93387-2

**Published:** 2021-07-07

**Authors:** Geoffroy Berthelot, Sonia Saïd, Vincent Bansaye

**Affiliations:** 1 Ecole Polytechnique, Centre de mathématiques appliquées (CMAP), 91128 Palaiseau, France; 2 REsearch LAboratory for Interdisciplinary Studies (RELAIS), 75012 Paris, France; 3 Institut national du sport, de l’expertise et de la performance (INSEP), 75012 Paris, France; 4 Office Français de la Biodiversité, Direction Recherche et Appui Scientifique, Unité Ongulés Sauvages-Unité Flore et Végétation, 01330 Birieux, France

**Keywords:** Ecology, Ecological modelling

## Abstract

Animal movement has been identified as a key feature in understanding animal behavior, distribution and habitat use and foraging strategies among others. Large datasets of invididual locations often remain unused or used only in part due to the lack of practical models that can directly infer the desired features from raw GPS locations and the complexity of existing approaches. Some of them being disputed for their lack of biological justifications in their design. We propose a simple model of individual movement with explicit parameters, based on a two-dimensional biased and correlated random walk with three forces related to advection (correlation), attraction (bias) and immobility of the animal. These forces can be directly estimated using individual data. We demonstrate the approach by using GPS data of 5 red deer with a high frequency sampling. The results show that a simple random walk template can account for the spatial complexity of wild animals. The practical design of the model is also verified for detecting spatial feature abnormalities and for providing estimates of density and abundance of wild animals. Integrating even more additional features of animal movement, such as individuals’ interactions or environmental repellents, could help to better understand the spatial behavior of wild animals.

## Introduction

Animals live in an environment that is patchy and hierarchical, and the manner in which individuals search for spatially dispersed resources is crucial to their success in exploiting them^1^. At the same time, the tracking of animals using the modern global positioning system (GPS) now allows for the collection of important datasets on animal locations^2^. They are often used for the analysis of the home range behavior, i.e. restrict their movements to self-limited portions of space far smaller than expected from their sole locomotion capacities^3^ and, more generally, to better understand the spatial and temporal behavior of animals^4,5^. New, smaller and reliable devices allow for gathering large datasets (e.g. locations or activity data for instance) at a finer temporal and spatial scale and offer a greater opportunity to investigate animal movement at the individual scale. However, datasets often remain only partially used due to both the lack of practical models that can directly infer the desired features from raw GPS locations and the complexity of existing approaches. Meanwhile, ecologists in particular are called to develop new capabilities to deal with these large datasets^6,7^.

The modeling of animal movement includes a wide range of methodologies: biased and/or correlated random walks (BCR)^8–11^, the disputed Lévy Flight/walk^12–16^, Stochastic Differential Equation (SDE)^17–20^ including diffusion models based on the two-dimensional Ornstein-Uhlenbeck process^21–25^, Hidden Markov Models (HMMs)^26–28^, state space models^29^, step-selection functions^30^ and other more exotic algorithms using *ad-hoc* rules to mimic movement features such as memory^31,32^. Lévy Flight has convenient patterns but ecological motivations are scarce^15^. SDE -the continuous analog of BCR—or the Brownian bridge and Movement Model^33^ may be used to interpolate the trajectory between two observations. SDE includes a drift (directional) and one or several random diffusion processes^20,34^. A comprehensive review on the modeling of individual animal movement underlines the complexity and lack of biological meaning in some of these methods^35^.

BCRs are simple and convenient tools to model animal movement as the discrete time is well adapted to regular GPS data^34^. The parameters of the BCR can be directly interpreted in terms of the behavior of the animal and the resulting movement pattern is directly related to the set of chosen parameters. In other state-switching models, the resulting pattern may instead be a composite of states that may not be biologically meaningful with regard to behavioural states^35^. BCR are tractable and flexible in their design and ecologists can use them to model a particular behavior, including parameters that correspond to the attraction of some locations, the inertia and memory feature of the movement, time dependence of the movement, local interactions with other individuals, etc. Some key features of animal movement have already been identified by previous studies, including diffusion (or randomness) which corresponds to an isotropic random motion, where the individual has the same probability to go in all directions; Attraction (directional bias) where the movement of the animal is anisotropic and is confined in an area or domain, according to^36^ and other studies^3^, while the attraction may depend on the distance from the isobarycenter of locations^37^; Inertia (correlated component) where the movement of the animal is also shaped by foraging tasks where the animal alternates exploration periods—the path has high tortuosity—with straightforward movements^38^. These three features can be implemented as parameters of a BCR.

More precisely, the advantages of using a BCR approach are:

- *parsimony*: the BCR dynamics are only depending on the set of parameters. They do not depend on additional or ad-hoc parameters. All the parameters can directly be inferred from the dataset,
- *interpretation*: each parameter can be directly interpreted in terms of animal behavior and ecology,
- *flexible design*: the number of parameters can be easily modified and the BCR can also be simulated in continuous or discrete time and space, depending on the process we wish to model,
- *well adapted to positioning data*: the approach is adapted to discrete time series such as positioning data and can be used independently of the time and spatial scales of the dataset. Thus, it can directly exploit the data without the requirement of any additional procedure for data preparation.

**Figure 1:**
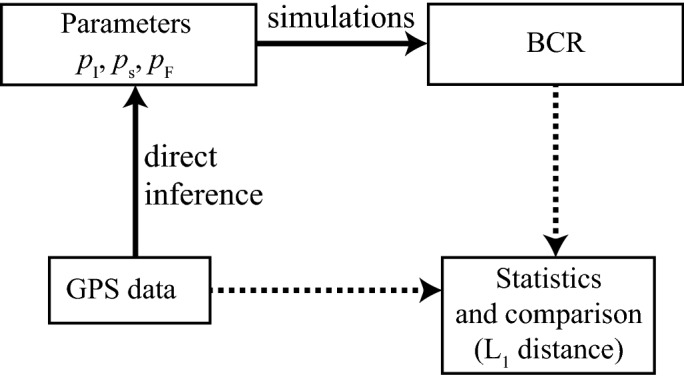
Framework used for testing the BCR model performance, for one animal. Black lines detail the two operations processed from the GPS dataset. The 3 parameters are estimated from the GPS data and—using these parameters—1000 simulations of the BCR model are computed. No particular operations are associated with the dotted black lines, but they show how the BCR and the GPS dataset are evaluated and compared using the statistics.

This study aims at modeling animal movement of sedentary individuals over short periods of approximatively 1 year^39^, without reproduction and in a homogeneous landscape using GPS data sets and a BCR. The model is implemented with the three aforementioned parameters and we use the GPS dataset of 5 red deer (*Cervus elaphus*) to demonstrate the approach (pictured in Fig. 1). We also study how the temporal resolution (i.e. the sampling rate) affects the estimations of the three parameters. We finally challenge this approach as a practical tool for inferring environmental and behavior information from the dataset.

## Methods

A simple methodological framework was established for testing the BCR model using empirical datasets, consisting of the GPS data of 5 animals. For each of these 5 animals, the three parameters were accordingly tuned using a straightforward estimation procedure. This procedure uses the empirical datasets to infer the parameters’values (Fig. 1). We also used the datasets to assess the model’s reliability—or performance-. We also detail other analyzes that were carried out to ensure the robustness and consistency of the approach, including the deterministic nature of the 5 statistics and a sensitivity analysis. This analysis consists in evaluating the performance of the BCR using a sweep method that produce arbitrary values of the parameters instead of using data-driven estimations. All BCR simulations and the five statistics were performed using MATLAB Version 7.13.0.564 (R2011b).

### Data

**Figure 2:**
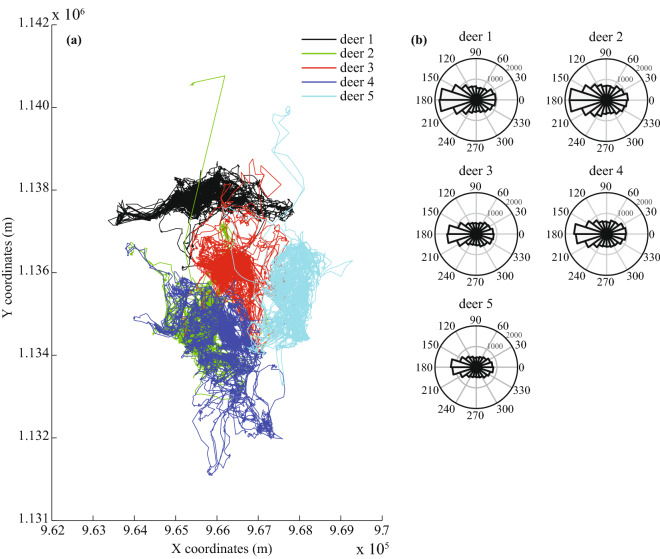
Individual paths of the five red deer. Individual paths of the five red deer. The individual paths are plotted for the five red deer (left panel, **a**) along with the distribution of the relative turning angles (degrees) in polar plots (right panel, **b**). An angular value of 0 consists in a straight motion from the previous location, while a relative turning angle of **180** $^{\circ }$ c corresponds to a turn back.

The locations of 5 GPS-collared red deer (*Cervus elaphus*) were gathered at La Petite Pierre National Hunting and Wildlife Reserve (NHWR), in north-eastern of France (48.8321 (Lat.) / 7.3514 (Lon.)). The reserve is an unfenced 2670 ha forest area characteristics by deciduous trees (mostly *Fagus sylvatica*) in the western part and by coniferous species (mostly *Pinus sylvestris* and *Abies alba*) in the eastern part in nature reserve surrounded by crops and pastures. It is located at a low elevation area of the Vosges mountain range, which rises up to 400 m a. s. l. The climate is continental with cool summers and mild winters (mean January and July temperatures of **1.4** and **19.6** $^{\circ }$C, respectively, data from Phalsbourg weather station, Meteo France, from 2004 to 2017). Three ungulate species are present and mainly managed through hunting in the NHWR: wild boar, red deer and roe deer. The present study focuses on female red deer for test model. A detailed overview of the landscape and surroundings is given in^40^. The GPS data had regular observation frequencies with high frequency sampling (Table 1). In the following text, we note $X_i = [X_i^{(1)}, X_i^{(2)}]$ the locations of the individual with $X_i \in {\mathbb {R}}^{2}$, $i=1,2,\ldots ,n$ and where $X_i^{(1)}$, $X_i^{(2)}$ represent the longitude and latitude respectively. We use $t_{i}$ ($t_1=0$) as the time elapsed between two successive locations $X_{i-1}$, $X_{i}$ and

1 $$\begin{aligned} {\overline{T}}= \dfrac{1}{n} \sum _{i=1}^{n} t_i \end{aligned}$$

as the average sampling time. The trajectory of the animal, or ‘path’, was interpolated using linear interpolation between each pair of recorded observations (Fig. 2 and detailed in Supplementary Methods (Eq. 21) and associated Graphic 2). It approximates the animal travels in straight lines at constant velocity between each pair of locations^41^. The attractor $X_F$ of one individual was estimated as the isobarycenter of all recorded locations:

2 $$\begin{aligned} X_F = \left[ \frac{1}{n}\sum _{i=1}^{n} X_i^{(1)}, \frac{1}{n}\sum _{i=1}^{n} X_i^{(2)} \right] \end{aligned}$$

**Table 1:** Data summary. For each animal, the total number of observations *n* is given along with the period of collection (date and time), the sampling rate ${\overline{T}}$ (i.e. the average time between 2 observations) (in min.) and corresponding standard deviation, total distance (in kilometers), total recording time (in days) and average speed *s* (in $10^{-2}$ m.s^-1^).

Animal	*n*	Period of collection	${\overline{T}}$ ±s.d. (min.)	Tot. distance (km.)	Time (d.)	*s* ($10^{-2}$ m s^-1^)
Deer 1	29,520	09/01/2010 00:00–11/08/2010 23:50	10.49 ±3.18	945.67	214.99	5.09
Deer 2	27,324	10/12/2009 00:00–24/06/2010 23:50	10.38 ±3.69	1030.97	196.99	6.06
Deer 3	23,301	16/03/2010 00:00–07/09/2010 23:41	10.88 ±8.67	876.33	175.99	5.76
Deer 4	24,735	22/01/2010 00:00–21/07/2010 23:51	10.54 ±5.16	898.38	180.99	5.74
Deer 5	21,451	16/01/2010 00:01–24/06/2010 07:31	10.69 ±10.11	785.93	159.31	5.71

### BCR model

The model aims at estimating the location at the next time step, given the actual location *X* at step *i*:

3 $$\begin{aligned} X_{i+1} = f \left( X_i \right) \end{aligned}$$

such that the function $f(\cdot )$ is assumed to be representative of the behavior of the animal on sufficiently large time scales. We considered one individual of a given species with no interaction and simulated its movement in continuous space and discrete time in 2 dimensions. The BCR includes 3 parameters coupled with isotropic diffusion:

- *Diffusion*: A random direction with uniform spatial distribution in a 2D plane,
- *Bias* ($p_F$): An increased probability to go to a fixed point named attractor^42^. This attractor was estimated as the isobarycenter of all recorded locations, defined as $X_F$ (Eq. 2). This yields a bias or advection parameter in the direction of $X_F$. We use the term ’attraction’ for the bias component of the BCR and the term ’den’ for the attractor. In the data set we study, the den is equivalent to the deer’s bunk.
- *Correlated component* ($p_I$): This parameter increases the probability to move forward, i.e. to perform one step in the direction of the previous step. This is equivalent to a short term bias in movement, when the animal has inertia. We refers to ’inertia’ for the correlated component,
- *Immobility* ($p_s$): We included this as a specific parameter and the movement is stopped for one step. This takes into account the absence of movement between a pair of locations. It can be accredited to technological limitations with the satellite telemetry due to a weak GPS signal strength, possibly due to natural elements: such as when the animal was standing underneath a rock or due to dense clouds, dust particles, mountains or flying objects, such as airplanes). However, this can also be part of the behavior of the animals, during specific times: sleep cycles or foraging for instance. We use $d_{\min }$ to denote this distance cutoff and set $d_{\min }=10$m which corresponds to the magnitude of the error typically found in GPS locations^43^. We also use $d_{\min }$ to encapsulate GPS error and peculiar ecological behavior, not associated with $p_I$ or $p_F$, that are beyond the scope of this study.

**Figure 3:**
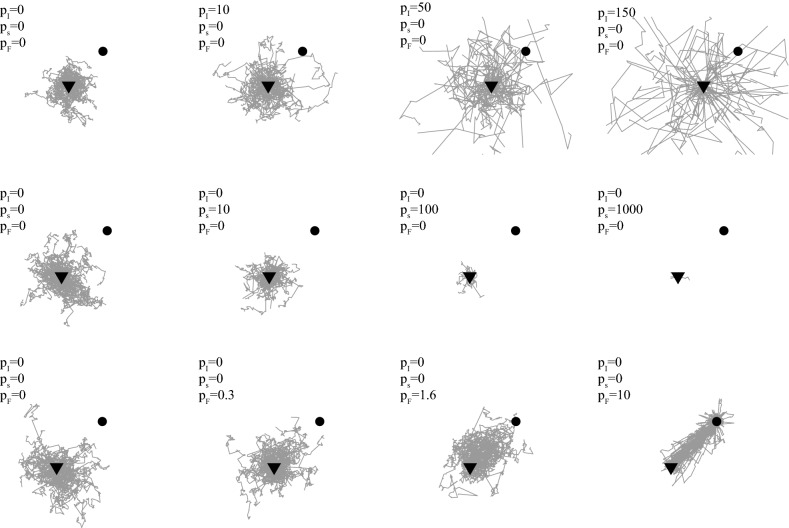
Simulated animal motions over arbitrary parameter values. Fifty motions of length $n_s=100$ steps are simulated and originate from a common centroid (downward-pointing triangle) with increased levels of correlation ($p_I$), immobility ($p_s$) and bias ($p_F$). Both the location of the attractor $X_F$ (black dot) and the log-normal parameters controlling the step size distribution are fixed ($\mu =3$, $\sigma ^2 = 1$).

The effect of each parameter is detailed in Fig. 3. The typical model contains all three parameters: $p_I$, $p_s$ and $p_F$ for describing animal motion while offering a trade-off between the number of parameters and the description of animal motion.

#### Estimation of the parameters

The estimation of the three parameters for each animal is based on the empirical datasets. We distinguished between the *states*, where one state is described by the pair $\left\{ X_{i-1}, X_i\right\}$ and the *situations*, where one situation is described by the past ($X_{i-1}$), current ($X_i$) and future ($X_{i+1}$) locations. Knowing both the state of the animal at a given time step *i* and its situation—the realization of movement at the next time step $i+1$—allowed for collecting the occurrences of inertia, immobilism and attraction. This could be done provided we account for the variability of the movement: the animal may not be heading exactly toward the den, or performing inertia with an exact angular value of $\pi$. Thus we discretized the space around the animal in 8 quadrants at each time step *i*. For example, if the animal was heading straight forward with a margin of $\pm \pi /8$ then it was considered in the situation of inertia. In other words, the state could fall in a situation of inertia with a margin of $\pm \pi /8$. Such a discretization can be represented as a matrix, depending on the state of the animal, its location and the location of the den at each time step (see Supplementary methods, Eq. 19). In order to gather enough data samples per situation, we arbitrary used angular thresholds of $\pi /8$ as a convenient trade-off between data scarcity and precision loss. Using smaller threshold values (say $\pi /10$) may result in too few samples per situations. Using larger threshold values such as $\pi /4$ may result in a loss of precision while capturing additional movement samples that may not correspond to the situation.

We first needed to define in which state is the animal at each step *i*. A state is the 2-tuple containing the previous and actual observation $\{X_{i-1}, X_i\}$. We wanted to distinguish between non-conflicting and conflicting states, where a non-conflicting state is when the animal is in one state only, while a conflicting state is when the animal is in two states at once. We defined two conflicting states:

4 $$\begin{aligned} {\mathscr {H}}_{IF}:=\{i : \widehat{ \left| X_{i-1} X_i X_F \right| } \le \pi /8\} \end{aligned}$$

when the animal was already heading toward the den $X_F$, and:

5 $$\begin{aligned} {\mathscr {H}}_{Is}:=\{i : d\left( X_{i-1}, X_i\right) \le d_{\min }\} \end{aligned}$$

when the distance between two consecutive observations was too small ($\le d_{\min }$ m.), describing an individual that was already immobile. Such that the subset of non-conflicting states is:

6 $$\begin{aligned} {\mathscr {H}}:=\{1,\cdots ,n\} - {\mathscr {H}}_{IF} - {\mathscr {H}}_{Is} \end{aligned}$$

We then needed to assess in which situation the animal was for each corresponding state. A situation is the 3-tuple $\left\{ X_{i-1}, X_i, X_{i+1}\right\}$. We defined three subsets of situations corresponding to a straight forward motion (*I*), no motion (*s*) and a motion toward the den (*F*):

7 $$\begin{aligned} I:= \{i: \pi - \pi /8 < \widehat{(X_{i-1} X_i X_{i+1})} \le \pi + \pi /8\} \end{aligned}$$

8 $$\begin{aligned} s:= \{i: d\left( X_i, X_{i+1} \right) \le d_{\min }\} \end{aligned}$$

9 $$\begin{aligned} F:=  \{i: \mid \widehat{X_{i-1} X_t X_{i+1}} - \widehat{X_{i-1} X_i X_F} \mid \le \pi /8\} \end{aligned}$$

With $d(\cdot ,\cdot )$ the Euclidean distance between two locations. For the situations in *s*, we considered that the animal is not performing a motion if the Euclidean distance between two successive locations was $\le d_{\min }$m.

We counted the number of states falling in each situation, for states in ${\mathscr {H}}$ (Eq. 6). We defined $x_{1}$, $x_{2}$, $x_{3}$ as the empirical proportion of cases corresponding to each situation:

10 $$\begin{aligned} {\left\{ \begin{array}{ll} x_1 = \dfrac{\# I \cap {\mathscr {H}}}{\# {\mathscr {H}}} ; \qquad x_1:=\dfrac{1+p_I}{\chi } \\[16pt] x_2 =\dfrac{\# s \cap {\mathscr {H}}}{\# {\mathscr {H}}} ; \qquad x_2:=\dfrac{p_s}{\chi }\\[16pt] x_3 = \dfrac{\# F \cap {\mathscr {H}}}{\# {\mathscr {H}}} ; \qquad x_3:=\dfrac{1+p_F}{\chi } \end{array}\right. } \end{aligned}$$

with $\chi = 8+p_{I}+p_{s}+p_{F}$. The values of $x_1$, $x_2$ and $x_3$ were then gathered for each animal. We did not use immobile locations (i.e. distances separating two successive observations must be $> d_{\min }$ m) for the estimations of $x_1$ and $x_3$. Solving Eq. (10) for $\chi$ with respect to $x_1$, $x_2$, $x_3$ yields:

11 $$\begin{aligned} \chi = \dfrac{6}{1-(x_{1} + x_{2} + x_{3})} \end{aligned}$$

Plugging in Eq. 10:

12 $$\begin{aligned} {\left\{ \begin{array}{ll} p_{I} = x_1 \chi -1\\ p_{s} = x_2 \chi \\ p_{F} = x_3 \chi -1 \end{array}\right. } \end{aligned}$$

Note that we assumed that $p_{IF} = p_I + p_F$ in ${\mathscr {H}}_{IF}$ and $p_{Is} = p_I + p_s$ in ${\mathscr {H}}_{Is}$ as a convenient arrangement and ignoring higher order conflicting cases. Investigating the step-size distribution in the 5 deers, we found a log-normal step size distribution (Supplementary Fig. S1). We then set a log-normal distribution $\ln {\mathscr {N}}(\mu , \sigma ^2)$ for the step size distribution for the step size in the BCR.

The same estimation procedure was used for configurations using a different number of parameters and quantity $\chi$ is accordingly calculated depending on the number of parameters used. It is possible to obtain negative values using this inference method. A parameter with a negative values reflects a direction that is not favored by the animal. In such a case, one should rethink the design of the BCR by changing the parameters (see Supplementary methods, section “negative parameters”). In the subsequent sections, we only consider parameters with positives values.

#### BCR dynamics

The BCR dynamics for each animal are completely determined by the three parameters $p_F$, $p_I$, $p_s$, taking values in ${\mathbb {R}}^{+}$, and the step-size distribution. If $p_F = p_I = p_s = 0$, the BCR resumes to a typical two-dimensional random walk with a log-normal step size distribution $\ln {\mathscr {N}}(\mu , \sigma ^2)$. The dynamics can be visualized in Fig. 3 for different values of each parameter. When simulating a step in the model, the motion in ${\mathscr {H}}$ is described by:

13 $$\begin{aligned} f \left( X_i \right) = {\left\{ \begin{array}{ll} \left\{ X_i^{(1)} + d \cos (\alpha _1) ; X_i^{(2)} + d \sin (\alpha _1) \right\} &{} \qquad \text {if } x \in [0,8[ \\ \left\{ X_i^{(1)} + d \cos (\alpha _2) ; X_i^{(2)} + d \sin (\alpha _2) \right\} &{} \qquad \text {if } x \in [8,8+p_I[ \\ X_i &{} \qquad \text {if } x \in [8+p_I, 8+p_I+p_s[ \\ \left\{ X_i^{(1)} + d \cos (\alpha _3) ; X_i^{(2)} + d \sin (\alpha _3) \right\} &{} \qquad \text {else} \end{array}\right. } \end{aligned}$$

with *x*, *d*, $\alpha _1$ random variables defined as $x \sim {\mathscr {U}} \in [0, \chi ]$, $d \sim \ln {\mathscr {N}}(\mu , \sigma )$, $\alpha _1 \sim {\mathscr {U}} \in [0,2\pi ]$. Variables $\alpha _2$, $\alpha _3$ are related to the angular values $\alpha _2 = {{\,{\mathrm{atan2}}\,}}(X_{i}^2 - X_{i-1}^2, X_{i}^1 - X_{i-1}^1)$, $\alpha _3 = {{\,{\mathrm{atan2}}\,}}(X_{F}^{(2)} - X_{i}^{(2)}, X_{F}^{(1)} - X_{i}^{(1)})$ with ${{\,{\mathrm{atan2}}\,}}(y, x)$ the four quadrant inverse tangent function (14):

14 $$\begin{aligned} {{\,{\mathrm{atan2}}\,}}(y, x) = {\left\{ \begin{array}{ll} \arctan \left( {\frac{y}{x}}\right) &{} x> 0,\\ \arctan \left( {\frac{y}{x}}\right) +\pi &{} x< 0{\text {, }}y \ge 0,\\ \arctan \left( {\frac{y}{x}}\right) -\pi &{} x< 0{\text {, }}y< 0,\\ +{\frac{\pi }{2}} &{} x=0{\text {, }}y > 0,\\ -{\frac{\pi }{2}} &{} x=0{\text {, }}y < 0,\\ 0 &{} x=0{\text {, }}y=0\text {.} \end{array}\right. } \end{aligned}$$

The motion in ${\mathscr {H}}_{Is}$ is:

15 $$\begin{aligned} f \left( X_i \right) = {\left\{ \begin{array}{ll} \left\{ X_i^{(1)} + d \cos (\alpha _1) ; X_i^{(2)} + d \sin (\alpha _1) \right\} &{} \qquad \text {if } x \in [0,8[ \\ X_i &{} \qquad \text {if } x \in [8, 8+p_I+p_s[ \\ \left\{ X_i^{(1)} + d \cos (\alpha _3) ; X_i^{(2)} + d \sin (\alpha _3) \right\} &{} \qquad \text {else} \end{array}\right. } \end{aligned}$$

The motion in ${\mathscr {H}}_{IF}$ is:

16 $$\begin{aligned} f \left( X_t \right) = {\left\{ \begin{array}{ll} \left\{ X_i^{(1)} + d \cos (\alpha _1) ; X_i^{(2)} + d \sin (\alpha _1) \right\} &{} \qquad \text {if } x \in [0,8[ \\ X_t &{} \qquad \text {if } x \in [8, 8+p_s[ \\ \left\{ X_i^{(1)} + d \cos (\alpha _2) ; X_i^{(2)} + d \sin (\alpha _2) \right\} &{} \qquad \text {else} \end{array}\right. } \end{aligned}$$

### Statistics for describing animal movement

We simulated $N=1000$ BCR and used 5 statistics to assess the model reliability on spatial features including: (*i*) the distribution of relative turning angles which provides information about the movement of the animal, (*ii*) the home range which provides information about the spatial density of observations and (*iii*) observation counts using still and mobile transects, providing information on absolute observation abundance^44^. A detailed description of each statistic is provided in Supplementary Methods and Fig. S2. The reliability—or performance—was assessed in each animal and studied statistic using two error terms $e_1$ and $e_2$. Error $e_1$ is the $L^1$ norm to compare the differences between the statistic ${\tilde{\mathscr {S}}}$ computed over a simulated path, and the statistic $\smash {{\mathscr {S}}}$ computed over the data-set:

17 $$\begin{aligned} e_1 \mathrel {\mathop :}= \sum {\text {errors}} = \sum _{k=1}^N |\smash {{\mathscr {S}}}- {\tilde{\mathscr {S}}}_k| \end{aligned}$$

With $k = 1,\ldots , N$ the number of simulations of the BCR. Error $e_1$ is the sum of absolute differences in the given statistic, and is a natural way of measuring the distance between the statistics computed on the data set and the trajectories generated using the BCR. We also focused on the average relative error $e_2$ as an indicator of the sensitivity:

18 $$\begin{aligned} e_2 \mathrel {\mathop :}= \dfrac{1}{N} \sum _{k=1}^N \dfrac{{\tilde{\mathscr {S}}}_k}{\smash {{\mathscr {S}}}} \end{aligned}$$

#### Distribution of turning angles

For each individual, the distribution of counter-clockwise relative turning angles $\widehat{(X_{i-1} X_i X_{i+1})}$ was gathered, provided $d(X_{i-1}, X_{i}) > d_{\min }$ and $d(X_{i}, X_{i+1}) > d_{\min }$. This means that we only kept the angles from observations that were separated by an Euclidean distance greater than $d_{\min }$.

#### Home range

We used an adaptive kernel density estimator (matlab package kde2d—kernel density estimation version 1.3.0.0) as an estimator of the utilization distribution^45^ to represent the home range of the animal. The approach of Z.I. Botev provided an estimate of observation density using a bivariate (Gaussian) kernel with diagonal bandwidth matrix^46^. The density was estimated over a grid of $210 \times 210$ nodes and we computed the home range area (in m^2^) for various values: 100, 99, 95, 90, 80, ..., 20, 10% of the estimated density. Similarly to the distribution of turning angles, we compared each value of the data’s home range against the simulated one.

#### Dilation

Dilation is generally used to account for the spatial attributes of an object such as to measure an area around the path or the volume of a brownian motion (see Wiener sausage^47^ and Gromov–Hausdorff distance). In our approach, we use dilation of both simulated and GPS paths for two reasons: to have a real—and comparable—number that accounts for how a trajectory has explored space and because it is natural tool from a census point of view (the dilated path corresponds to the area where the animal can be detected). Each simulated or real path was plotted in binary format in a window and dilated with a disk shape. The window size was set to a huge value in order to encapsulate the dilated path while preventing boundary effects, i.e. the convex envelope of the dilated area did not collide with any window border. We then estimated the surface covered by the dilated path for 100 different sizes of the disk, from disk size 1 to disk size 100. We compared each value of the data’s estimated surface against the simulated one.

#### Immobile transects

We used still transects that counted the number of times the animal was seen in their line of sight. We arbitrary set the line-of-sight value at 200 m. The number of sightings of each transect was gathered and ordered in decreasing order, thus breaking the spatial dependence. We then compared the bins of the resulting histogram in the data and in the simulated path.

#### Mobile transects

First, the movement of the animal was linearly interpolated from the GPS data, meaning that between two recorded locations the individual followed a linear path. The speed of the animal between two locations was accordingly reconstructed using the recorded times $t_i$ between each location. Second, we used mobile transects as the ecological sampling method, where each transect ‘count’ the intersection between its path and the animal’s one. The mobile transects followed a predefined path at a given constant speed as time increased. The area of vision of each transect was defined as a circle of a given radius. Each time the path of an individual collided with an area of vision, the count of the corresponding transect increased by 1. Two types of movements were used: linear and clockwise rotational transects. The initial locations of both types of transects are $X_1$ and $X_F$. Both the animal and mobile transects started to move at the same time. At each of the two locations $X_1$, $X_F$, 8 linear transects moved in the 8 cardinal directions, totalizing 16 transects. For the linear transects, every 10,000 time steps, we set $2 \times 8$ new transects starting at the same locations and following the same directions. Clockwise rotational transects were rotated around $X_1$ and $X_F$ using a 500 m radius. When we reached $t_n$, we gathered the total count (i.e. the count of all transects). For the two types of transects, we gathered the total count for 6 different lines of sight: 50, 100, 200, 400, 500, 1000 m. and 4 speeds: *s*/4, *s*/2, *s*, 2 *s* with *s* the average speed of the animal. We then aggregated the overall count in each of the two types of transects, and compared the results from the data and the simulated path (Supplementary Methods and Fig. S2).

### Scale invariance

Several authors pointed out that the temporal resolution of the discretization is of importance: it should be relevant to the considered behavioral mechanisms^5,48–50^. Schlägel and Lewis focused on the quantification of movement models’ robustness under subsampled movement paths^49^. They found that increased subsampling leads to a strong deviation of the central parameter in resource selection models^49,51^. They underlined that important quantities derived from empirical data (e.g. parameters estimates, travel distance or sinuosity) can differ based on the temporal resolution of the data^49,51^. Moreover, Postlethwaite and Dennis highlighted the difficulty of comparing model results amongst tracking-datasets that vary substantially in temporal grain^50^). Each of the studied dataset has a relatively high sampling rate (roughly 10 m) and a period of study that is appropriate to the analysis of animal movement at the year scale (Table 1). In order to investigate such a possible effect on the BCR dynamic, we changed the sampling rate of the movement path to ensure that the three parameters $p_I$, $p_s$ and $p_F$ are scale invariant. The movement path formed by the GPS observations $X_i$ was subsampled (decimated) for each individual. We only kept every $k^{\text {th}}$ observation starting with the first one and $k \in \left[ 1,10\right]$. For $k=1$ the path corresponded to the original one. The time spent between each successive observation was also accordingly reconstructed in order to keep track of ${\overline{T}}$ in subsampled movement paths. The time between two locations $X_i$ and $X_{i+k}$ was reconstructed as:

19 $$\begin{aligned} t_{j}' = \sum _{i=j}^{i+(k-1)} t_i \end{aligned}$$

with $j \in \left[ 1, 1+k, 1+2k, \ldots , n-\left( k-1 \right) \right]$. We did not change the value of $d_{\min }$ as we subsampled the movement path because we designed $p_s$ for capturing GPS noise and movements that are associated with peculiar ecological behaviors that are beyond the scope of this study in terms of time and spatial scales (foraging for instance). We then compared the resulting parameters $p_I$, $p_F$ and $p_s$ as the resampling rate *k* increased.

### Fluctuations

Whereas the BCR is a stochastic process, the deterministic aspects of the 5 statistics were tested with an increasing number of steps $n_s$. The statistic associated with each realization of the model (a simulated path) is a random variable. If the distribution of these random variables has low concentration (high variance) then it is not a convenient statistic as it cannot be used as a reference for assessing the model’s performance, even when averaging over multiples realizations. On the opposite, if the statistic is deterministic (no fluctuations) it can provide a reliable tool to assess the model’s performance. This was numerically tested over a range of increasing $n_s$ values with $n_s = 10^4, 2\times 10^4, \ldots , 4 \times 10^5$. For each of those step values, a set of 100 BCR was simulated with parameters $p_I$, $p_F$ and $p_s$ estimated from the first deer (see Table 2) and we studied the variance of the statistics.

### Sensitivity analysis

In order to assess whether the estimated parameters are optimal (i.e. providing the best possible performance) and to study parameter scarcity, we also evaluated the performance of the model using arbitrary weight values. We first started by evaluating how using one parameter instead of the three could alter the performance of the model. We then extended this sensitivity analyse by drawing arbitrary values for each parameter from a multi-dimensional square mesh, whose center corresponds to the estimated values of $p_I$, $p_s$, $p_F$, estimated using GPS data (Fig. 1). We additionally used values that are distant from the estimated ones, up to $p_I=3$, $p_F=3$ and $p_s=5$. We tested a total of 151 new configurations with these arbitrary values. For each configuration, we ran 150 simulations and evaluated them using the 5 statistics. The mean error of $|\smash {{\mathscr {S}}}- {\tilde{\mathscr {S}}}_k|$ and its standard deviation are gathered and plotted for each arbitrary configuration. As a resume, we replicate the framework described in Fig. 1 but we inject arbitrary parameters instead of using data-driven parameterisations.

### Application

The proposed model could be used to infer environmental and behavior information from the dataset. We chose to illustrate such an application by trying to detect anomalous voids (or holes) in the spatial territory of the individual using the GPS dataset and Monte-Carlo simulations of the model. Anomalous means that the observed void is not related to the randomness of the movement, but rather related to a geographical artifact. The parameters $p_I$, $p_F$, $p_s$, $\mu$ and $\sigma ^2$ of the BCR were accordingly estimated from the data of each individual, similarly to previous experiments (Fig. 1). A simple heuristic was used to find voids in empirical and simulated paths for each individual: we computed the alpha shape of all locations using a fixed alpha radius of 60 m. This allowed for determining the surface covered by all locations while preserving the voids. We then collected the area of each void provided they had an area of at least 100 m^2^. We focused on voids near the center of the alpha shape in order to avoid artificial voids, generated by the weak density of locations at the boundaries. We ran 10,000 iterations of the model for each animal and estimated the probability $p_{\varnothing }$ of finding voids of different sizes in the simulated paths. This probability was then compared to voids found in the GPS datasets and available environmental information was used to determine whether any geographical element(s) could explain the unexpected voids.

## Results

We found that the parameters $\mu$ and $\sigma ^2$ were close for all individuals (Table 2), and estimates of the three parameters for individuals 3 and 4 were similar. The values of $p_I$ and $p_F$ showed that inertia and attractor play a greater role in the movement of deer 5 ($p_I=0.22$, $p_F=0.24$), compared to the 4 other individuals. On the opposite, the movement of Deer 1 is characterized by immobilism ($p_I=0.01$, $p_s=2.01$ $p_F=0.01$). The immobility $p_s$ was stable across the individuals while $p_I$ and $p_F$ varied together (Table 2). The latter is a mechanistic effect, as they act as opposite forces.

**Table 2:** Estimated parameters. For each deer, the estimated parameters $p_I$, $p_F$, $p_s$ and the two parameters $\mu$, $\sigma$ that control the step size distribution are given.

Animal	$p_I$	$p_F$	$p_s$	$\mu$	$\sigma$
Deer 1	0.01	0.01	2.01	2.94	1.01
Deer 2	0.06	0.13	1.44	3.15	0.97
Deer 3	0.12	0.05	1.70	3.07	1.04
Deer 4	0.10	0.06	1.52	3.10	0.98
Deer 5	0.22	0.24	1.66	3.03	1.06

### Evaluation of the BCR model

**Figure 4:**
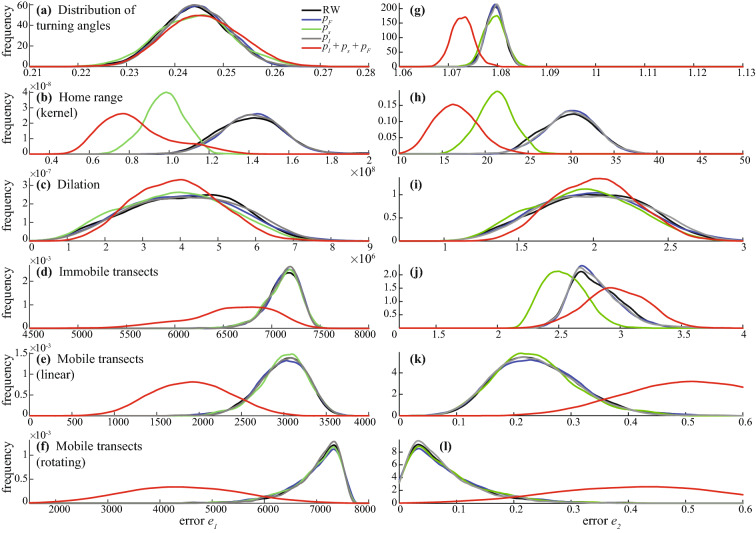
Density of error $e_1$ and $e_2$ of all 5 configurations tested in each statistic for deer 5. Left panels (**(a)**, **(b)**, **(c)**, **(d)**, **(e)**, **(f)**) correspond to error $e_1$ and right panels (**(g)**, **(h)**, **(i)**, **(j)**, **(k)**, **(l)**) to error $e_2$. The statistics are given for the distribution of turning angles (**(a)**, **(g)**), home range (**(b)**, **(h)**), dilation (**(c)**, **(i)**), immobile transects (**(d)**, **(j)**), and mobile transects (**(e)**, **(k)**) and (**(f)**, **(l)**). Smallest absolute errors $e_1$ should be close to 0 to indicate a good performance of the model, while relative errors $e_2$ should be close to 1 to indicate a good performance. Densities are fitted by the Epanechnikov kernel function. All the errors for all red deers are presented in Supplementary Fig. S3.

The distribution of errors $e_1$ and $e_2$ in the 5 statistics is provided in Fig. 4 for deer 5 and in Supplementary Figure S3 for all 5 deers. The table containing the mean error and standard deviation, median error and interquartile range for all deer and for error $e_1$ is also provided in Supplementary file S2. For all deers, we showed that combining the parameters plays an important role in modeling deer behavior. Configurations with only one parameter did not perform well on average while further investigations showed that combining $p_I$, $p_F$ and $p_s$ allows for a better description of movement, especially regarding the census statistics for both linear and rotational transects and home range estimates (Supplementary Figure S3 and Table S2).

### Scale invariance

**Figure 5:**
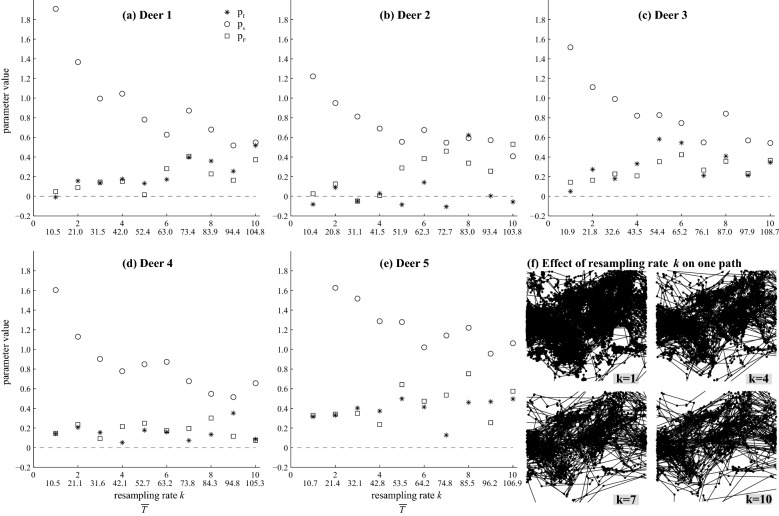
Scale invariance. The values of $p_I$, $p_F$ and $p_s$ are estimated with increasing resampling (or decimation) rate *k* for each deer: panels **(a)**–**(e)**. The first X-axis corresponds to the resampling rate *k* while the second X-axis is ${\overline{T}}$, the average sampling time. An example of resampling is presented in panel **(f)**, with *k* increasing from $k=1$ (upper left sub panel) to $k=10$ (lower right sub panel).

The parameters $p_I$ and $p_F$ remain almost constant with increased subsampling (Fig. 5). On the other hand, distance-related parameters such as $p_s$, $\mu$, $\sigma ^2$ are highly sensitive to the resampling rate *k*. This is a mechanistic effect of the subsampling procedure: as we increase *k*, the distance between each pair of GPS locations increases, resulting in less observations falling in the state *s* (Eq. 8) which in turn results in smaller $p_s$.

### Fluctuations

We found that the variance decreases or does not change as the number of simulated steps increases in most of the statistics (Fig. S4). Thus, 4 of the 5 statistics are robust and add limited randomness to the results when the number of steps increases. The long-term trend is not clear in the mobile transects case as we investigated the variance over $4\times 10^5$ steps and we may only observe a transient increase or stagnation. This statistic is expected to be similar to the one of immobile transects but the speed of convergence to the null variance may be very slow and it may take a much larger number of steps. The variance of the estimated areas in the dilation statistic increases with $n_s$ because we dilated the simulated paths in a huge window, encapsulating the whole path including a very large portion of empty space around it. This was done to prevent boundary effects when assessing the area of dilated paths: to make sure that dilated paths do not hit any of the window bounds. Otherwise this would produce biased, underestimated areas. However, using a smaller window or, again, a much larger number of steps would result in a null-variance.

### Sensitivity analysis

We showed that using the 3 parameters instead of one provides better results (Fig. 4 and S3). The inferred values for the single parameter configuration of the BCR are given in table S1and corresponding mean, standard deviation, median and interquartile range are presented in table S2. When investigating arbitrary values for the three parameters, we show that the empirically inferred estimates provide better performance in all deers. It means that the BCR simulations that use the three parameters with values directly inferred from the dataset show better performance in average (Fig. S5). This is clear in the distribution of turning angles, where using arbitrary values leads to an increase of the mean error. While some arbitrary values may produce better performance, they only work for selected individuals and specific statistics. For instance, configuration 140 with arbitrary parameters values $\left\{ p_I=3, p_s=4, p_F=1 \right\}$ provide better results in all statistics except the distribution of turning angles for deer 1. The inferred parameters values for deer 1 are $\left\{ 0.01,2.01,0.01\right\}$, such that one could conclude that an arbitrary increase of $p_I$ and $p_F$ will produce better performance (to the exception of reproducing the correct turning angles distribution pattern) for all deers and statistics. However, using the parameters values of configuration 140 produce highest mean error in the distribution of turning angles, home range (Kernel) estimate and dilation for deer 4. Moreover, even a small change in the values of a successful configuration with arbitrary parameters can lead to complete different results in a given animal and statistics.

One leading observation is that a small increase in immobilism $p_s$ -0.2 or 0.4 for instance—yields better results in most statistics for all deers (supporting Fig. S5). This is true provided the two other parameters $p_I$ and $p_F$ are not far from the inferred values. However, artificially increasing $p_s$ always lead to failure in reproducing the correct angular distribution. An arbitrary increase of $p_F$ also reduces the mean error in the 3 statistics which summarize the aggregated spatial localizations (i.e. kernel estimates, dilation and fixed transects) but ultimately fails in providing accurate results in mobile transects.

### Application: detecting spatial voids

**Figure 6:**
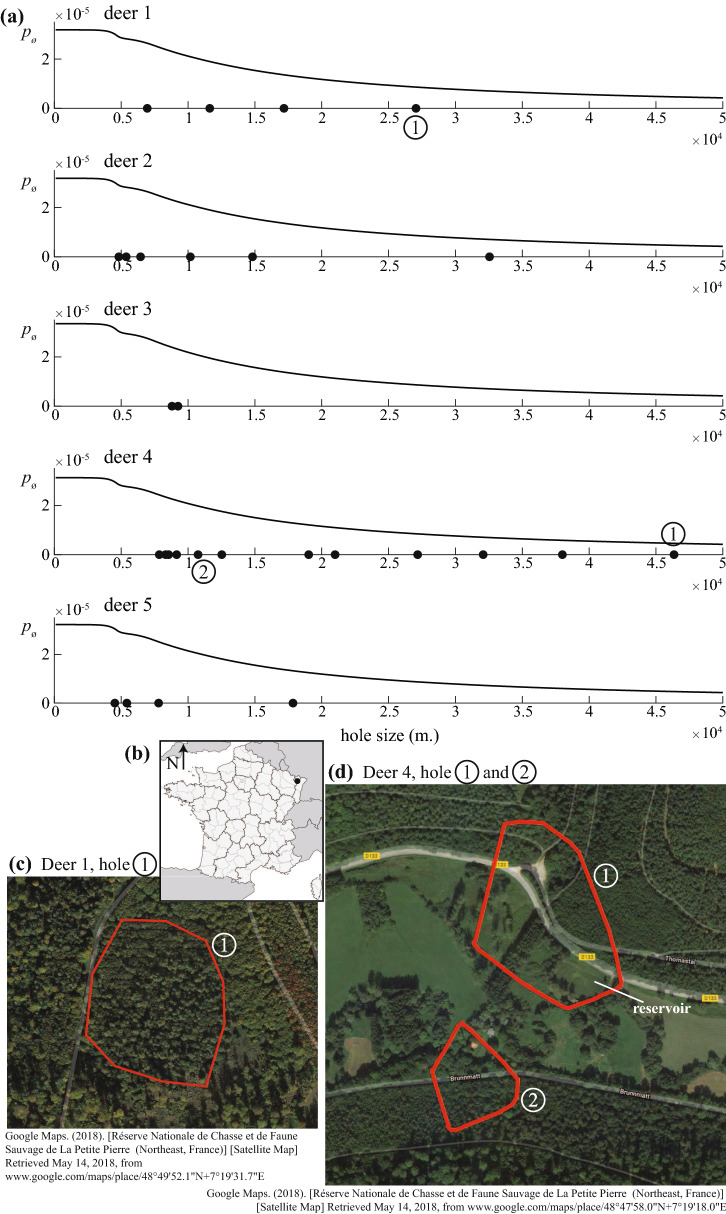
Using the framework and model to identify spatial voids in movement paths. In panel (**a**) the probability $p_{\varnothing }$ of finding voids of different sizes in the simulated paths is given for each deer (black lines). All voids $>100\,\text {m}^2$ detected in the empirical GPS location are given (black dots in x-axis). Selected voids (circled numbers) correspond to voids that both are near the center of the alpha shape and have a low $p_{\varnothing }$ (see alpha shape figures in Supplementary S6). (**b**) Geographic location of the study area (black dot). The three voids detected in deer 1 and 4 are detailed in panel (**c**) and (**d**) along with the environmental features. Image in (**b**) was created by TomKr and is distributed under GNU Free Documentation License. It corresponds to the map of France with regions and departments in equirectangular projection and was realized with free IGN data base GeoFla (http://www.ign.fr).

The resulting alpha shapes and detected voids (holes) are presented for each deer in Supplementary Fig. S6. The probability $p_{\varnothing }$ of observing such voids is computed and showed in Fig. 6. Many voids whose area fell in the interval $\left[ 0, 1.5\times 10^4\right]$ were related to boundary conditions, where the alpha shape produced artificial voids due to less dense areas. However, the Monte Carlo simulations show that 3 voids, located inside the alpha shape (void 1 (deer 1) and voids 1 and 2 (deer 4)), should not appear. In other words, these voids are possibly not related to movement randomness but to other spatial features, with good probability. The distributions of errors for each configuration varied in each deer.

## Discussion

In this work we aim at providing valuable and reliable ecological information regarding the components of animal movement. We introduce a simple and tractable model to deal with animal movement, based on a two-dimensional BCR in discrete time and continuous space that allows for combining the ecological forces in a simple way. The parameters of the BCR are directly inferred from the GPS data recorded in a large herbivore and its performance is assessed in 5 spatial and ecologically-related statistics. Four of them differ from the typical signals or parameters calculated based on empirical relocations^52^ and address the home range size, census issues and animal behavior. While there is no methodological consensus in the most appropriate way to check for similarity of individual movement paths, turning angles and home-range can provide concurrence in movement parameters over time without including positional information^53,54^. In addition, census information give an aggregated information, based on the intersections of movement paths with either mobile or fixed transects. The framework is presented in Fig. 4 and explains how synthetic (or simulated) paths parameterized from empirical observations are ultimately compared to the empirical paths throughout the statistics. However, we emphasize that the attributes of the data (such as the sampling rate for instance) should be consistent amongst different individuals in order to allow for inter-individual comparison^50^. It is also important to have a sufficient number of locations as precision in parameter estimation scales with the sample size, meaning that the more locations, the higher the precision.

We focus on three essential forces that allow for an efficient description of animal motion over large periods of time. The results display that by combining those parameters, we get a much better description of animal movement compared to an unbiased random-walk with a log-normal step size distribution. Results also show that using three parameters produce accurate results in describing animal movement compared to using a single parameter. This confirms that animal movement is a complex process, driven by several forces instead of a single and dominant one. Both the absolute error $e_1$ and mean relative errors $e_2$ do provide the same results, showing that the BCR model is robust in terms of the norm ($e_1$) and in sensitivity of the norm ($e_2$). An additional analyze was conducted to further test if the parameters inferred from the data set provide the best performance. Results show that configurations with an increase in immobilism $p_s$ and—to lesser extent—den attraction $p_F$ values can improve the performance of the model. However, such an arbitrary increase comes at the cost of a lesser performance in describing the distribution of turning angles or estimating mobile transects. Thus, the configuration using the parameters estimated from the data set does provide the more balanced performance in all statistics for all deers amongst the 152 configurations tested.

The inertia $p_I$, describing the short-term memory effect, is the first force introduced in this approach. Whether the use of land space by the animal is dependent on short-term or long-term memory is a debated topic. It gave rise to a series of studies that emphasized the importance of memory in animal movement from a biological or modeling perspective^55–61^. These studies also underlined that inferring memory effects directly from relocations is not a trivial task. Those relocations instead depend on a mixture of effects, including landscape and territorial constraints, resource patches and possibly long-term memory. Using a single memory feature $p_I$ might be a too simple approximation for efficiently capturing the memory effect. In our approach it is possible to alter $p_I$ in order to include several previous steps instead of just one.

Immobility $p_s$ combines several features of animal movement including animal at rest, in vigilant state, and GPS noise. Multiple factors are known to affect GPS noise, including topographic exposure, canopy cover, vegetation height and the slow movement of the ionosphere. The latter changes by a few centimeters during 30sec intervals^2^, possibly introducing up to 20 fold this bias in each of the recorded GPS observations. However, this is small regarding the average step size of non-immobile movements, ranging from 41 m (red deer 1) to 46 m (red deer 3 and 5). Thus, we assume that the measured step lengths and turning angles reflect the reality. Immobile (i.e. $\le d_{\min }$) observations represent a large proportion in our total datasets: **25.0%** (red deer 1), **17.2%** (red deer 2),** 22.0%** (red deer 3), **19.0%** (red deer 4) and **23.6%** (red deer 5), associated with specific behaviors such as on-site foraging, eating, resting, etc. The estimates of $p_s$ in all five animals are greater than inertia or attractor (Table 2), underlining the importance of considering immobility when analyzing the movement of red deer. This is in line with previous experimental studies that showed the high frequency of feeding, resting cycles in red deer and labile diet^62^.

Site fidelity is the recurrent visit of an animal to a previously occupied location. This is a well-known and wide-spread behavior in the animal kingdom^63^. The animal favors locations that are ecologically valuable and related to a foraging or explorative behavior. In our approach, we rather and simply depict site fidelity using one single attractor $p_F$. The fact it improves the performance of the model when combined with inertia and immobilism confirms that site fidelity (or a simplified estimation of it) should be taken into account when modeling deer movement.

The model can be sophisticated by including more complicated environmental aspects of individual movement, such as spatial memory^58,59^, reinforcement and site fidelity^64^, environmental predictability^65^ including landscape effect^66^, interacting individuals and prey-predator dynamics. Bailey and Codling had shown that turning angle distributions are affected by the underlying movement behaviours^67^ and that using one amalgamated distribution to analyse all movement is not effective^9,68^. In this work, we used 3 parameters and a single uniform distribution in all directions (diffusion) to account for other underlying or more subtle behaviors—foraging, etc.-. Similarly, we used a single log-normal distribution to describe the step-size distribution. The BCR model can be adapted to include more parameters that would result in a less diffusive movement, and multiple distributions can be used to model the step-size at different and smallest time scales. Distributions and parameters values can also be nested such that the model can adapt to different time and spatial scales.

One leading rational of this work is to investigate the model’s ability to address ecological challenges such as estimating the abundance of a given species (census) or detecting anomalous spatial features. We use both mobile and immobile simulated transects to illustrate how the model could be used in the first problem. The probability of counting the same animal multiple times can then be estimated using Monte Carlo simulations. The second issue is, for instance, to detect spatial voids in empirical movement paths. The location of animals may present empty spatial voids (or holes) of various sizes. This may be related to environmental conditions such as urban areas, water, cliff, or other ecological reasons (such as interactions with other individuals $\textit{e.g.}$ repulsive marks) and other factors. Using numerical simulations of the model, we are able to detect anomalous voids in the dataset, that are not related to randomness but to human activity. The void 1 (deer 1) reveals that some environmental changes took place between the recording time of the GPS location in 2010 and the satellite image in 2018. After cross-checking with additional information from the OFB, we learned that the identified area was a forest enclosure. This explains why the deer was not able to reach this area. Both voids 1 and 2 of deer 4 also are related to human activities: forest roads, buildings and one artificial reservoir.

This work introduces a tractable model, based on a two-dimensional BCR for describing animal movement in discrete time and continuous space. The model allows for a direct and explicit estimation of the three parameters that provide the optimal design regarding the studied statistics. Moreover, it allows for deriving reliable (i.e. independent from the GPS sampling rate) and quantitative information about the components of animal movement. Results show that combining the parameters is a key component in modeling movement, while allowing for an accurate description of the turning angles, home range size and census issues. The model also has practical applications for addressing ecological issues such as census or spatial anomalies. While we only focus on 5 animals to demonstrate the approach, the model is general and can be applied to any other species. We considered only one attractor per animal in the proposed approach and both the existence and influence of multiple attractors are yet to be investigated. Additional behavior features such as the spatial reinforcement, memory of *n* previous steps, activity rhythms (such as the circadian cycle), distance from the attractor, landscape/habitat effect^66^, interactions with other animals and topological issues are currently being investigated and will be included in a future work. Comparing the proposed method to other existing approaches, such as the HMMs, would allow for evaluating its benefits and disadvantages in terms of both precision (error) and computational resources. In particular, the scale invariance of the parameters may provide a benefit compared to other scale-dependent approaches. An additional improvement would be to consider $d_{\min }$ as an average speed cutoff instead of a distance cutoff. This would allow for capturing immobile or slower movements independently from the average sampling time ${\overline{T}}$. Another point of interest is the development of a continuous version of the proposed model, where the direction of the step is drawn from a specific distribution, whose parameters are yet to be empirically characterized.

## Supplementary Information

Supplementary Figure 1

Supplementary Figure 2

Supplementary Figure 3

Supplementary Figure 4

Supplementary Figure 5

Supplementary Figure 6

Supplementary Methods

Supplementary Table 1

Supplementary Table 2
