# Supplementary material for: A random walk model that accounts for space occupation and movements of a large herbivore
Source: Sci Rep. 2021 Jul 7;11:14061. doi: 10.1038/s41598-021-93387-2 (PMC8263821; doi:10.1038/s41598-021-93387-2)
Supplement: Supplementary file 1 — Supplementary Figure 1 [file 41598_2021_93387_MOESM1_ESM.pdf]

A random walk model that accounts for space occupation and movements of a large herbivore

Geoffroy Berthelot[1,2,3], Sonia Saïd[4], and Vincent Bansaye[1]
1 Ecole Polytechnique, Centre de mathématiques appliquées (CMAP), Palaiseau, 91128, France
2 REsearch LAboratory for Interdisciplinary Studies (RELAIS), Paris, 75012, France
3 Institut national du sport, de l'expertise et de la performance (INSEP), Paris, 75012, France
4 Office Français de la Biodiversité, Direction Recherche et Appui Scientifique, Unité Ongulés Sauvages-Unité Flore et Végétation, Birieux, 01330, France

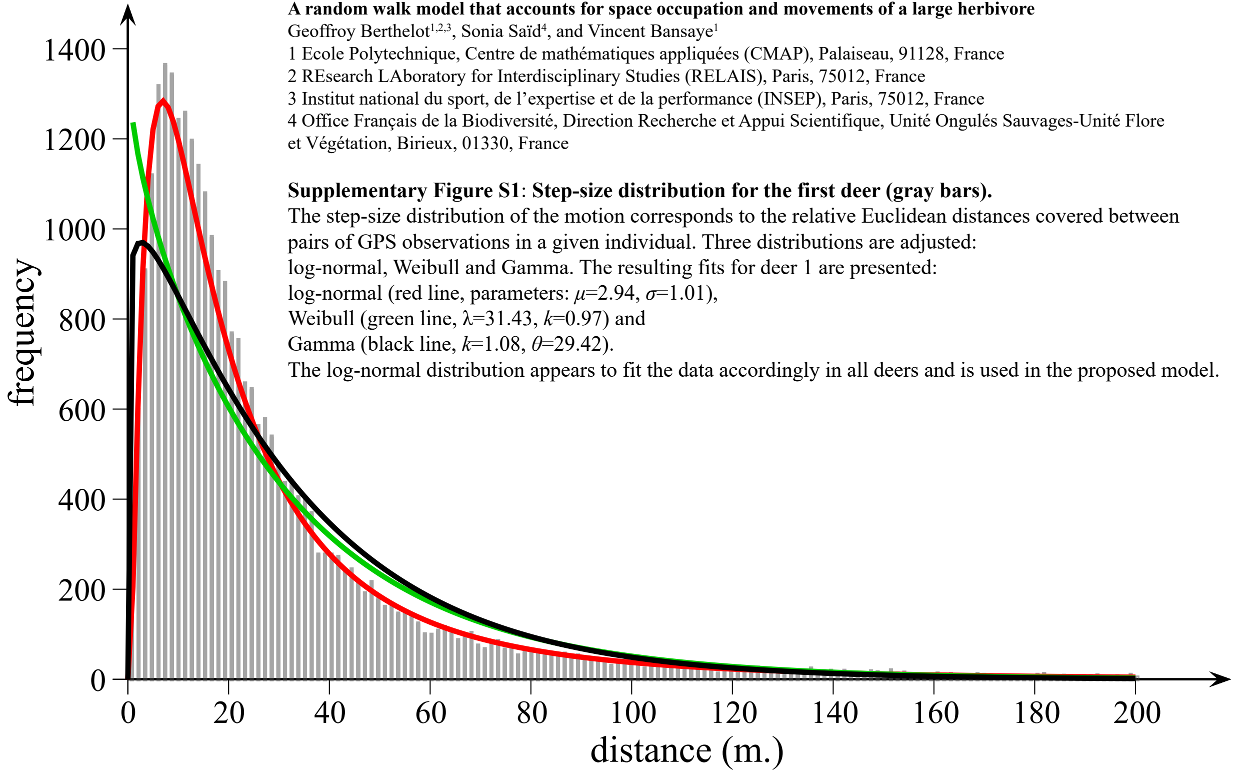

Supplementary Figure S1: **Step-size distribution for the first deer (gray bars).**
The step-size distribution of the motion corresponds to the relative Euclidean distances covered between pairs of GPS observations in a given individual. Three distributions are adjusted:
log-normal, Weibull and Gamma. The resulting fits for deer 1 are presented:
log-normal (red line, parameters: μ=2.94, σ=1.01),
Weibull (green line, λ=31.43, k=0.97) and
Gamma (black line, k=1.08, θ=29.42).
The log-normal distribution appears to fit the data accordingly in all deers and is used in the proposed model.
