# Supplementary material for: A random walk model that accounts for space occupation and movements of a large herbivore
Source: Sci Rep. 2021 Jul 7;11:14061. doi: 10.1038/s41598-021-93387-2 (PMC8263821; doi:10.1038/s41598-021-93387-2)
Supplement: Supplementary file 2 — Supplementary Figure 2 [file 41598_2021_93387_MOESM2_ESM.pdf]

A random walk model that accounts for space occupation and movements of a large herbivore

Geoffroy Berthelot[1,2,3], Sonia Saïd[4], and Vincent Bansaye[1]

1 Ecole Polytechnique, Centre de mathématiques appliquées (CMAP), Palaiseau, 91128, France

2 REsearch LAboratory for Interdisciplinary Studies (RELAIS), Paris, 75012, France

3 Institut national du sport, de l'expertise et de la performance (INSEP), Paris, 75012, France

4 Office Français de la Biodiversité, Direction Recherche et Appui Scientifique, Unité Ongulés Sauvages-Unité Flore et Végétation, Birieux, 01330, France

Supplementary Figure S2

Details of the 5 statistics. For each GPS data of deer 1 (left panels), a statistic is computed. Error estimates for error e_1 are detailed in the right panels.

STATISTICS	ERROR ESTIMATES

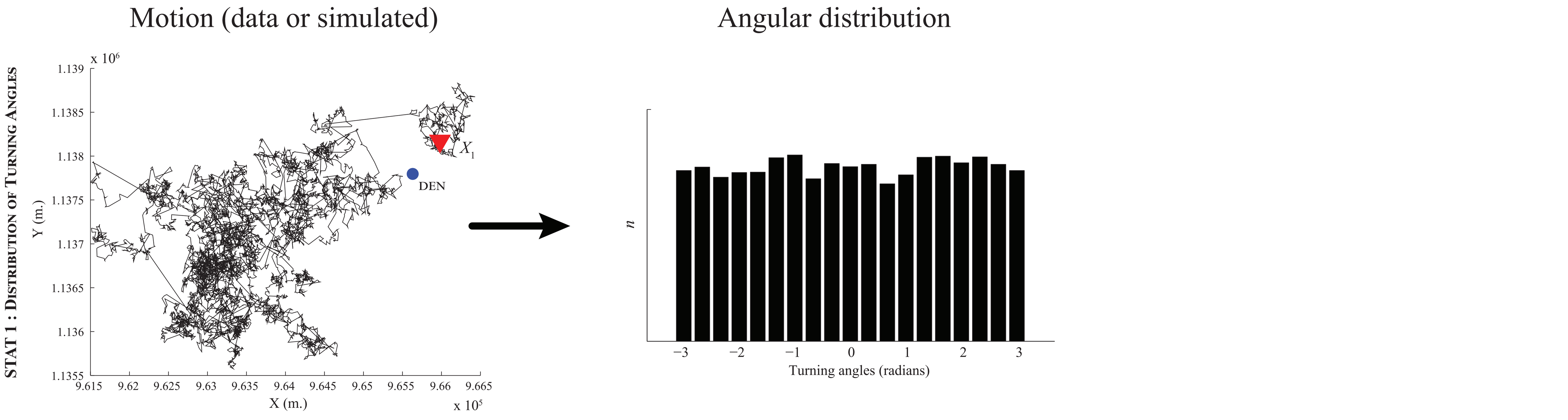

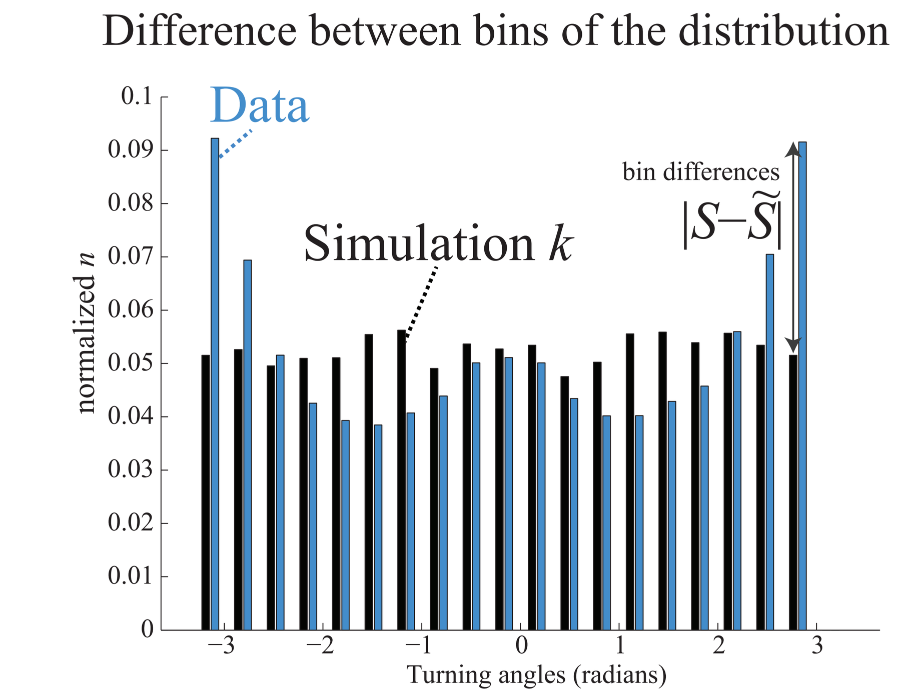

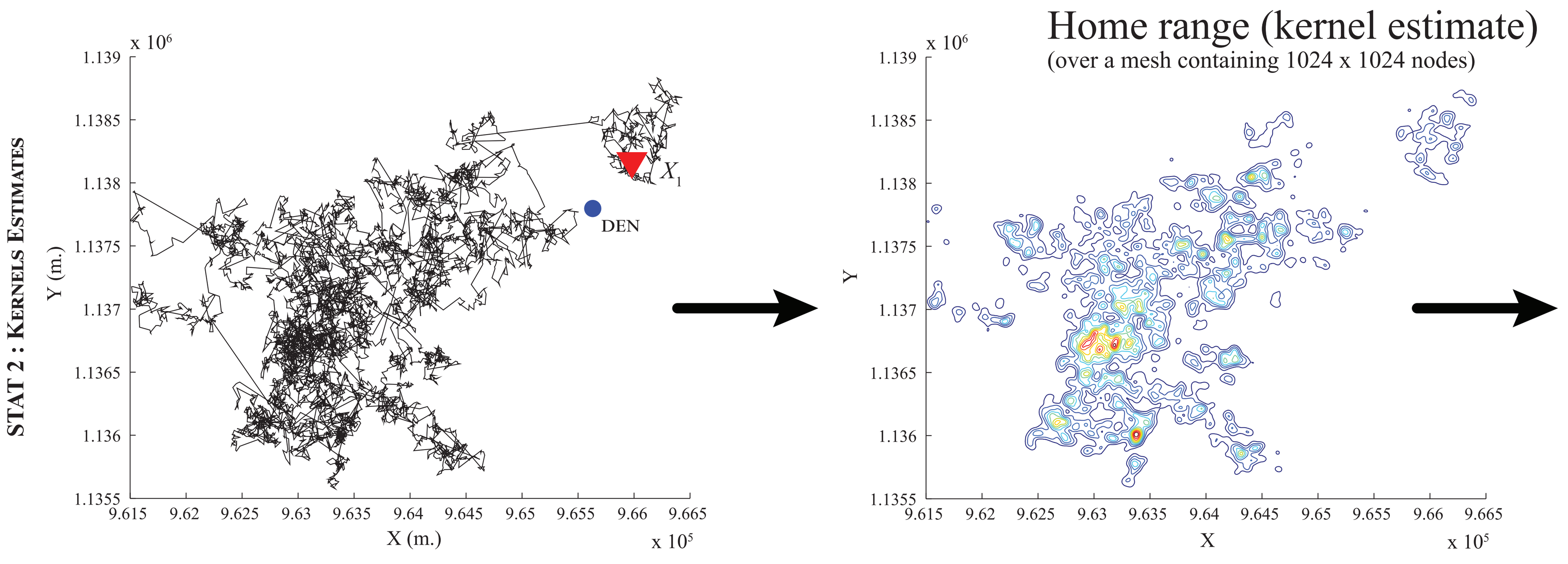

Collect the n most dense nodes, representing X%* of the estimated kernel, then estimate the area covered by these n nodes

*X = [10, 20, 30, ..., 100]

Difference between estimated areas A for each X%:

X (%)		10	20 ...	100
A_{data}	(10^6 m²)	0.0074	0.0250 ...	2.0331
$A_{simulation\ k}$	(10^7 m²)	0.0127	0.0310 ...	3.6114
$\|S-\tilde{S}\|$	(10^7 m²)	0.0120	0.0285 ...	3.4081

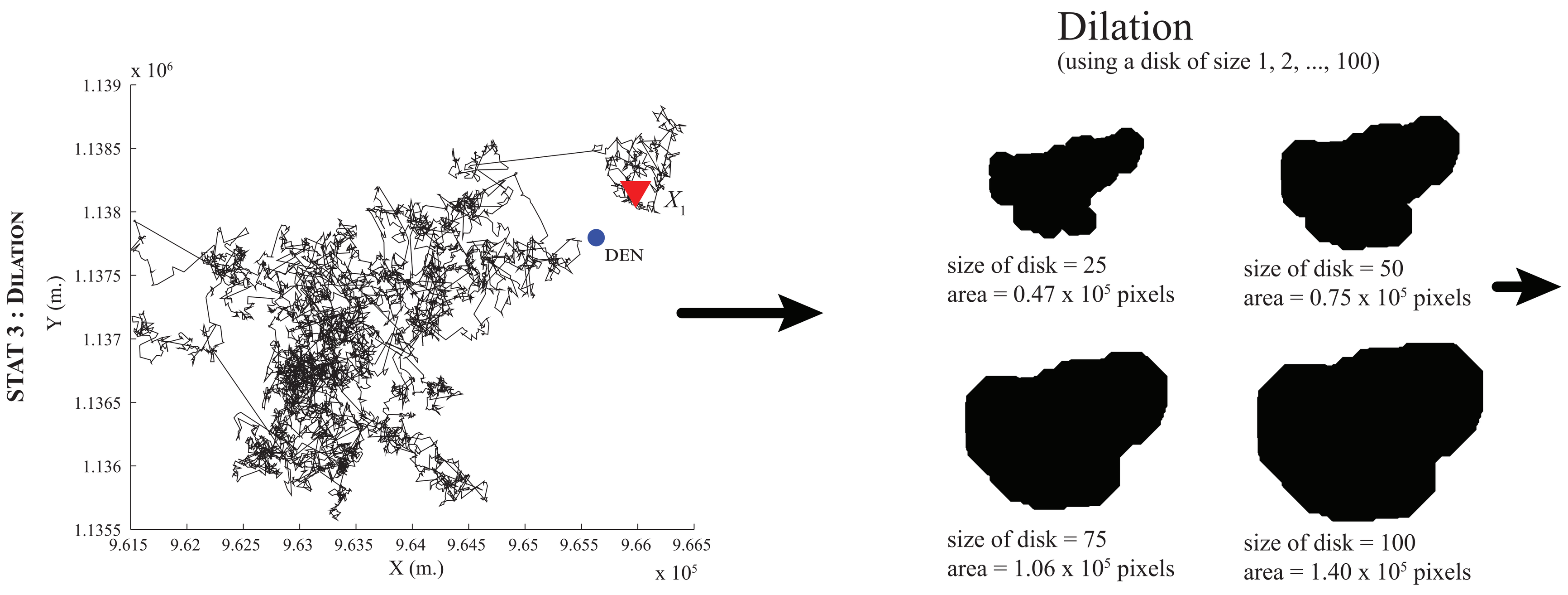

Collect the estimated areas for all sizes of the disk

Difference between estimated areas A:

size of disk (pixels)		...	25	... 50	... 100
A_{data}	(10^5 pixels)	...	0.4080 ...	0.6577 ...	1.2655
$A_{simulation\ k}$	(10^5 pixels)	...	0.4717 ...	0.7488 ...	1.4027
$\|S-\tilde{S}\|$	(10^4 pixels)	...	0.6366 ...	0.9103 ...	1.3716

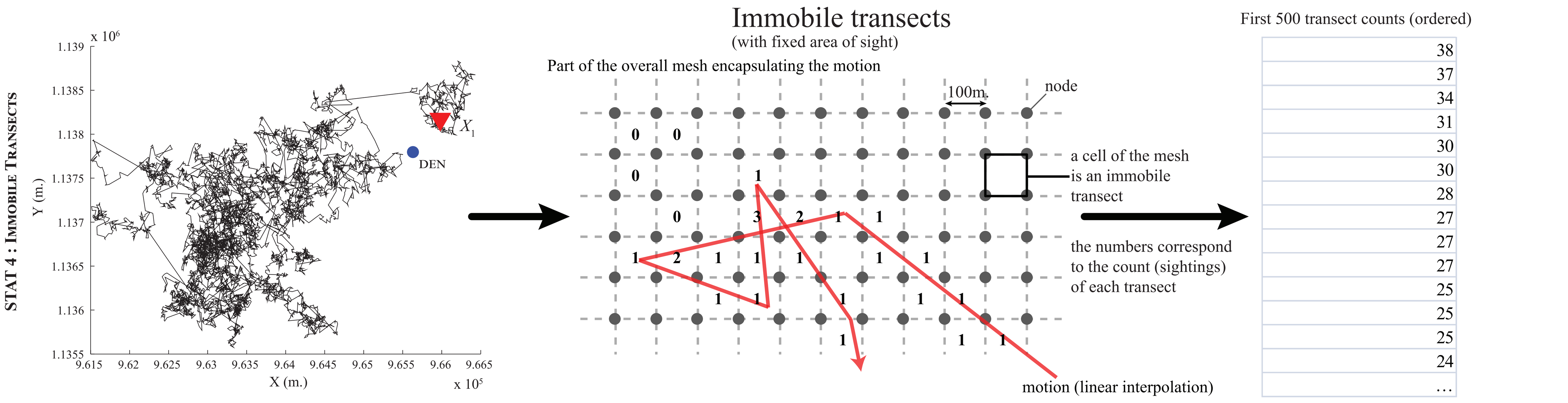

Difference between first 500 transect counts (ordered)

data	simulation k	$\|S-\tilde{S}\|$
388	38	350
380	37	343
309	34	275
258	31	227
227	30	197
225	30	195
...	...	...

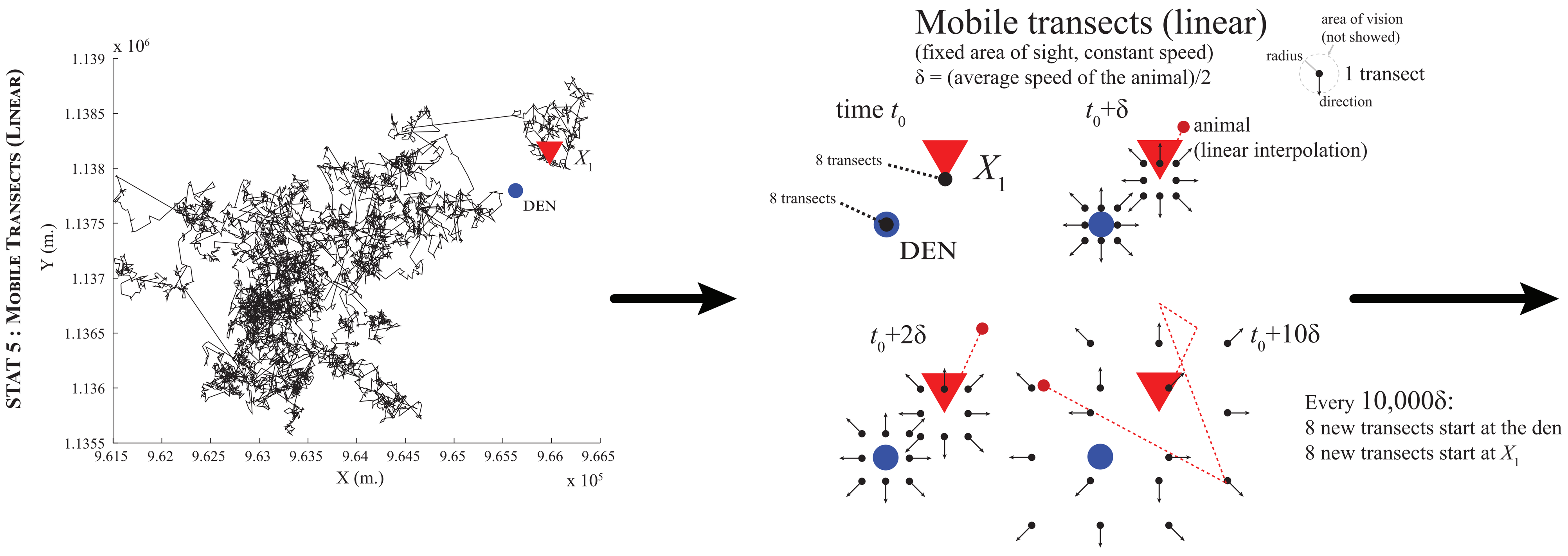

For each speed and radius, all transects counts are aggregated

Difference between transect counts for all radius and speeds:

$|S-\tilde{S}|$ = |Total count (data) - total count (simulation k)|

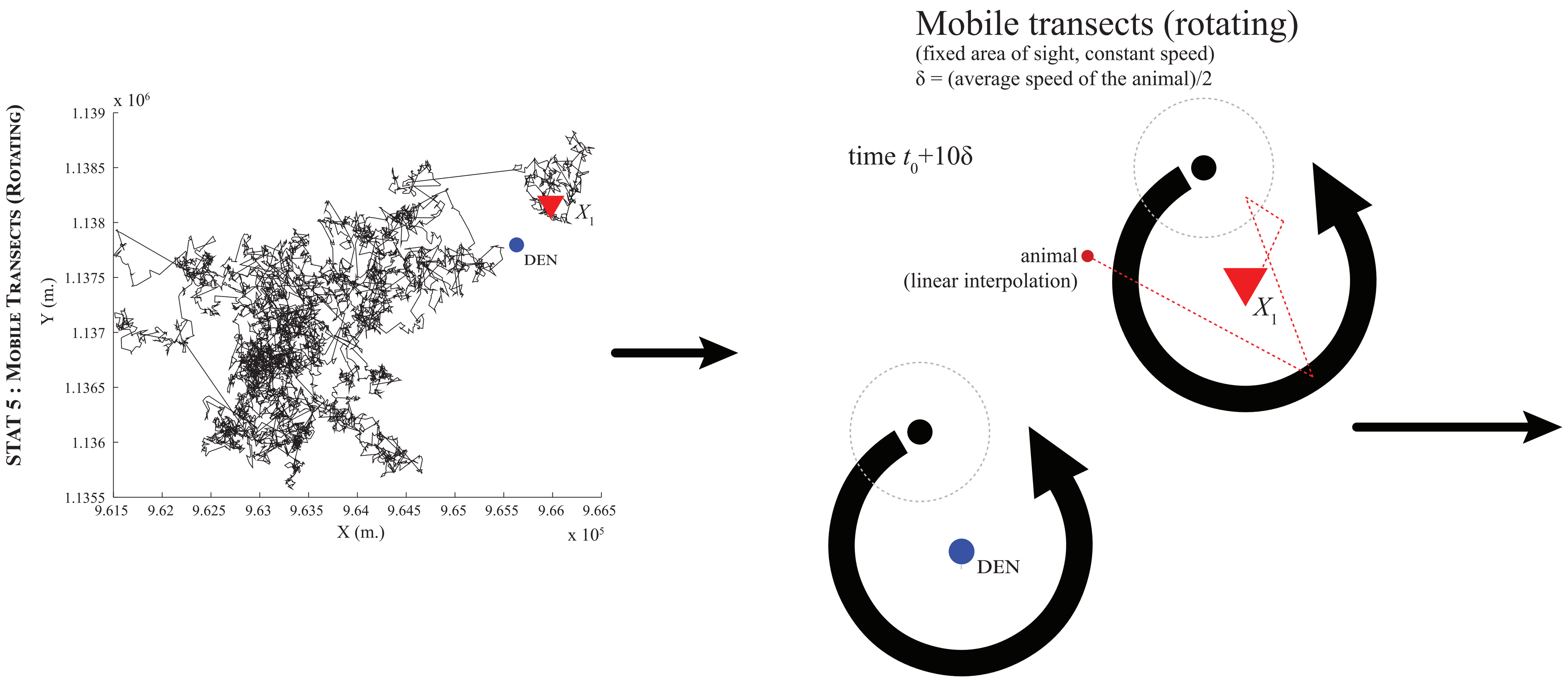

For each speed and radius, the two transects counts are aggregated

Difference between transect counts for all radius and speeds:

$|S-\tilde{S}|$ = |Total count (data) - total count (simulation k)|
