# Supplementary material for: A random walk model that accounts for space occupation and movements of a large herbivore
Source: Sci Rep. 2021 Jul 7;11:14061. doi: 10.1038/s41598-021-93387-2 (PMC8263821; doi:10.1038/s41598-021-93387-2)

A random walk model that accounts for space occupation and movements of a large herbivore

Geoffroy Berthelot[1,2,3], Sonia Saïd[4], and Vincent Bansaye[1]
1 Ecole Polytechnique, Centre de mathématiques appliquées (CMAP), Palaiseau, 91128, France
2 REsearch LAboratory for Interdisciplinary Studies (RELAIS), Paris, 75012, France
3 Institut national du sport, de l'expertise et de la performance (INSEP), Paris, 75012, France
4 Office Français de la Biodiversité, Direction Recherche et Appui Scientifique, Unité Ongulés Sauvages-Unité Flore et Végétation, Birieux, 01330, France

Supplementary Figure S4

Variance of the statistics with an increasing number of simulated steps n_s, from $n_s=10^4$ to $n_s=4\text{x}10^5$. We perform additional simulations in the $[10^4, 2\text{x}10^4]$ interval to show the rapid decrease in the home range statistic (**b**). We also show the intra-class variance for all 100 disks sizes in the dilation (**c**) and the intra-bins variance for each of the 500 bins of each distribution in immobile transects (**d**).
The variance of the normalized count in mobile transects are detailed for all 4 speeds and 8 radiuses tested for mobile transects (**e** and **f**).

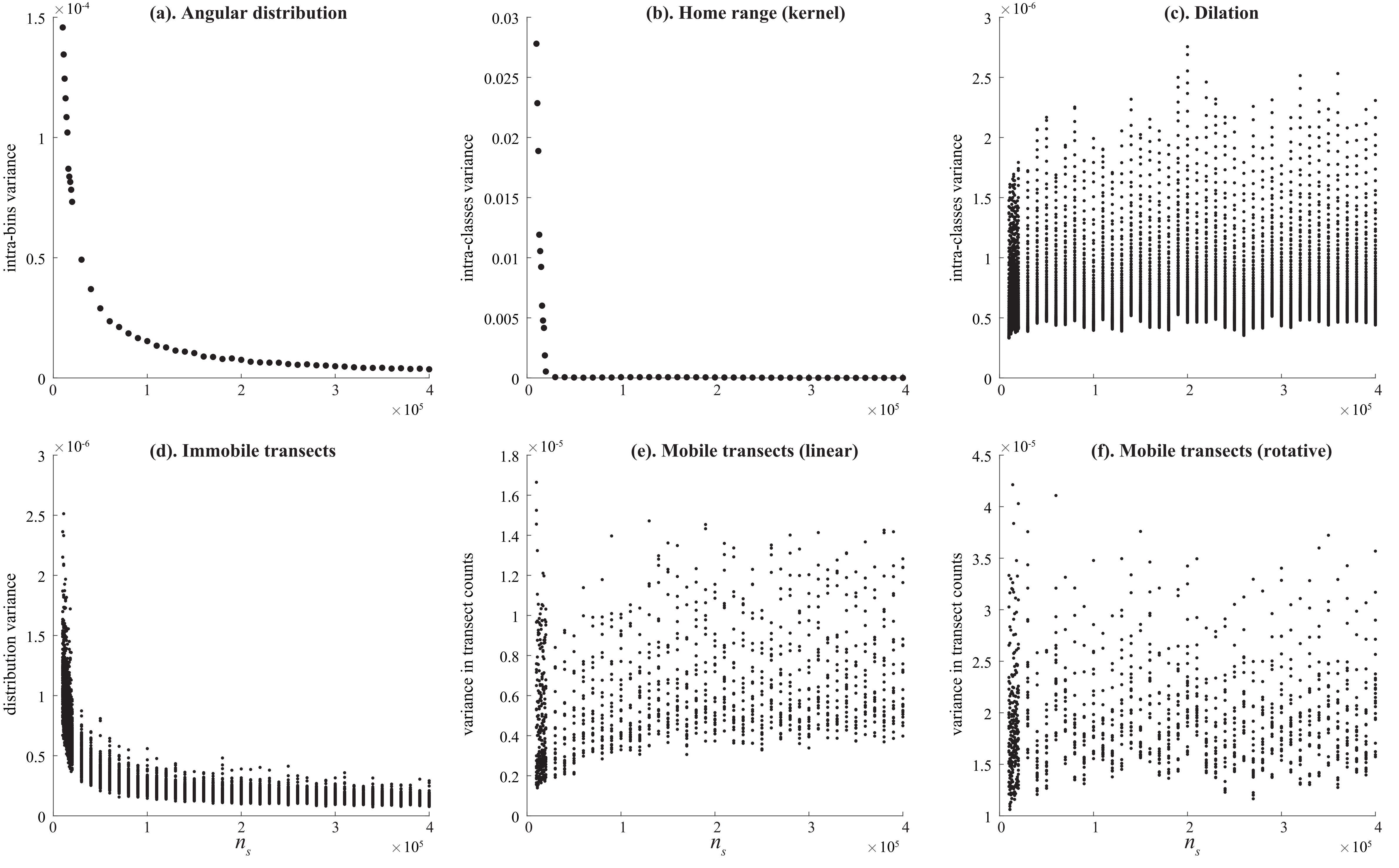

Supplement: Supplementary file 4 — Supplementary Figure 4 [file 41598_2021_93387_MOESM4_ESM.pdf]
