# Supplementary material for: A random walk model that accounts for space occupation and movements of a large herbivore
Source: Sci Rep. 2021 Jul 7;11:14061. doi: 10.1038/s41598-021-93387-2 (PMC8263821; doi:10.1038/s41598-021-93387-2)
Supplement: Supplementary file 5 — Supplementary Figure 5 [file 41598_2021_93387_MOESM5_ESM.pdf]

A random walk model that accounts for space occupation and movements of a large herbivore
Geoffroy Berthelot[1,2,3], Sonia Saïd[4], and Vincent Bansaye[1]
1 Ecole Polytechnique, Centre de mathématiques appliquées (CMAP), Palaiseau, 91128, France
2 REsearch LAboratory for Interdisciplinary Studies (RELAIS), Paris, 75012, France
3 Institut national du sport, de l'expertise et de la performance (INSEP), Paris, 75012, France
4 Office Français de la Biodiversité, Direction Recherche et Appui Scientifique, Unité Ongulés Sauvages-Unité Flore et Végétation, Birieux, 01330, France

Supplementary Figure S5: **Additional configurations of the BCR model.**
Arbitrary values of p_I, p_s and p_F against inferred parameters. A total of 151 new configurations are tested against the parameters inferred from GPS data. The mean (black line) error and standard deviation (gray shaded area) of the inferred parameters are presented in each panel. The mean (black dots) error and standard deviation (black lines) is given for each of the 151 configurations in each panel. The x-axis is the configuration index and the detail of each configuration is reported in large tables per index.
The first index corresponds to the inferred parameters and each new configuration is ordered by Euclidean distance ('distance') from the inferred parameters. For each of the new configuration, 150 simulations are performed.

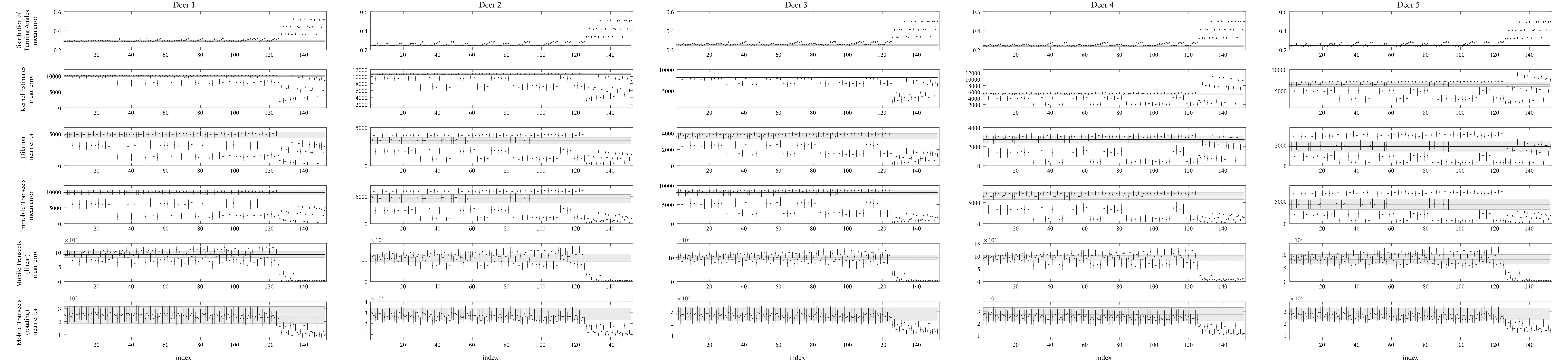

Index correspondante table (each index corresponds to a configuration with arbitrary weights):

Deer 1

index	1	2	3	4	5	6	7	8	9	10	11	12	13	14	15	16	17	18	19	20	21	22	23	24	25	26	27	28	29	30	31	32	33	34	35	36	37	38
distance	0.00	0.20	0.20	0.20	0.20	0.20	0.20	0.28	0.28	0.28	0.28	0.28	0.28	0.28	0.28	0.28	0.28	0.28	0.28	0.35	0.35	0.35	0.35	0.35	0.35	0.35	0.35	0.40	0.40	0.40	0.40	0.40	0.40	0.45	0.45	0.45	0.45	0.45
pI	0.01	0.01	0.01	-0.19	0.01	0.01	0.21	-0.19	0.01	0.01	0.21	-0.19	0.01	0.01	0.21	-0.19	-0.19	0.21	0.21	-0.19	-0.19	0.21	0.21	-0.19	-0.19	0.21	0.21	0.01	0.01	-0.39	0.01	0.01	0.41	-0.39	-0.19	-0.19	0.01	0.01
ps	2.01	2.21	1.81	2.01	2.01	2.01	2.01	2.21	2.21	2.21	2.21	1.81	1.81	1.81	1.81	2.01	2.01	2.01	2.01	2.21	2.21	2.21	2.21	1.81	1.81	1.81	1.81	1.61	2.41	2.01	2.01	2.01	2.01	2.21	1.61	2.41	1.61	1.61
pF	0.01	0.01	0.01	0.01	-0.19	0.21	0.01	0.01	-0.19	0.21	0.01	0.01	-0.19	0.21	0.01	-0.19	0.21	-0.19	0.21	-0.19	0.21	-0.19	0.21	-0.19	0.21	-0.19	0.21	0.01	0.01	0.01	-0.39	0.41	0.01	0.01	0.01	0.01	-0.19	0.21

index	39	40	41	42	43	44	45	46	47	48	49	50	51	52	53	54	55	56	57	58	59	60	61	62	63	64	65	66	67	68	69	70	71	72	73	74	75	76
distance	0.45	0.45	0.45	0.45	0.45	0.45	0.45	0.45	0.45	0.45	0.45	0.45	0.45	0.45	0.45	0.45	0.45	0.45	0.45	0.49	0.49	0.49	0.49	0.49	0.49	0.49	0.49	0.49	0.49	0.49	0.49	0.49	0.49	0.49	0.49	0.49	0.49	0.49
pI	0.01	0.01	0.01	0.01	0.21	0.21	0.41	-0.39	0.01	0.01	0.41	-0.39	-0.39	-0.19	-0.19	0.21	0.21	0.41	0.41	-0.39	-0.39	-0.19	-0.19	-0.19	-0.19	-0.19	-0.19	0.21	0.21	0.21	0.21	0.21	0.21	0.41	0.41	-0.39	-0.39	-0.19
ps	2.21	2.21	2.41	2.41	1.61	2.41	2.21	1.81	1.81	1.81	1.81	2.01	2.01	2.01	2.01	2.01	2.01	2.01	2.01	2.21	2.21	1.61	1.61	2.21	2.21	2.41	2.41	1.61	1.61	2.21	2.21	2.41	2.41	2.21	2.21	1.81	1.81	1.81
pF	-0.39	0.41	-0.19	0.21	0.01	0.01	0.01	0.01	-0.39	0.41	0.01	-0.19	0.21	-0.39	0.41	-0.39	0.41	-0.19	0.21	-0.19	0.21	-0.19	0.21	-0.39	0.41	-0.19	0.21	-0.19	0.21	-0.39	0.41	-0.19	0.21	-0.19	0.21	-0.19	0.21	-0.39

index	77	78	79	80	81	82	83	84	85	86	87	88	89	90	91	92	93	94	95	96	97	98	99	100	101	102	103	104	105	106	107	108	109	110	111	112	113	114
distance	0.49	0.49	0.49	0.49	0.49	0.57	0.57	0.57	0.57	0.57	0.57	0.57	0.57	0.57	0.57	0.57	0.57	0.60	0.60	0.60	0.60	0.60	0.60	0.60	0.60	0.60	0.60	0.60	0.60	0.60	0.60	0.60	0.60	0.60	0.60	0.60	0.60	0.60
pI	-0.19	0.21	0.21	0.41	0.41	-0.39	-0.39	0.01	0.01	0.01	0.01	0.41	0.41	-0.39	-0.39	0.41	0.41	-0.39	-0.39	-0.39	-0.39	-0.39	-0.39	-0.19	-0.19	-0.19	-0.19	0.21	0.21	0.21	0.21	0.41	0.41	0.41	0.41	0.41	0.41	-0.39
ps	1.81	1.81	1.81	1.81	1.81	1.61	2.41	1.61	1.61	2.41	2.41	1.61	2.41	2.01	2.01	2.01	2.01	1.61	1.61	2.21	2.21	2.41	2.41	1.61	1.61	2.41	2.41	1.61	1.61	2.41	2.41	1.61	1.61	2.21	2.21	2.41	2.41	1.81
pF	0.41	-0.39	0.41	-0.19	0.21	0.01	0.01	-0.39	0.41	-0.39	0.41	0.01	0.01	-0.39	0.41	-0.39	0.41	-0.19	0.21	-0.39	0.41	-0.19	0.21	-0.39	0.41	-0.39	0.41	-0.39	0.41	-0.39	0.41	-0.19	0.21	-0.39	0.41	-0.19	0.21	-0.39

index	115	116	117	118	119	120	121	122	123	124	125	126	127	128	129	130	131	132	133	134	135	136	137	138	139	140	141	142	143	144	145	146	147	148	149	150	151	152
distance	0.60	0.60	0.60	0.69	0.69	0.69	0.69	0.69	0.69	0.69	0.69	1.71	2.43	2.43	2.43	2.98	2.98	2.98	3.30	3.30	3.30	3.45	3.72	3.72	3.73	3.73	3.73	3.73	4.11	4.11	4.11	4.34	4.34	4.34	4.67	4.67	4.67	5.18
pI	-0.39	0.41	0.41	-0.39	-0.39	-0.39	-0.39	0.41	0.41	0.41	0.41	1.00	1.00	2.00	1.00	2.00	1.00	2.00	1.00	3.00	1.00	2.00	2.00	3.00	1.00	3.00	1.00	2.00	2.00	3.00	2.00	3.00	1.00	3.00	3.00	2.00	3.00	3.00
ps	1.81	1.81	1.81	1.61	1.61	2.41	2.41	1.61	1.61	2.41	2.41	3.00	3.00	3.00	4.00	3.00	4.00	4.00	3.00	3.00	5.00	4.00	3.00	3.00	4.00	4.00	5.00	5.00	4.00	4.00	5.00	3.00	5.00	5.00	4.00	5.00	5.00	5.00
pF	0.41	-0.39	0.41	-0.39	0.41	-0.39	0.41	-0.39	0.41	-0.39	0.41	1.00	2.00	1.00	1.00	2.00	2.00	1.00	3.00	1.00	1.00	2.00	3.00	2.00	3.00	1.00	2.00	1.00	3.00	2.00	2.00	3.00	3.00	1.00	3.00	3.00	2.00	3.00

Deer 2

index	1	2	3	4	5	6	7	8	9	10	11	12	13	14	15	16	17	18	19	20	21	22	23	24	25	26	27	28	29	30	31	32	33	34	35	36	37	38
distance	0.00	0.20	0.20	0.20	0.20	0.20	0.20	0.28	0.28	0.28	0.28	0.28	0.28	0.28	0.28	0.28	0.28	0.28	0.28	0.35	0.35	0.35	0.35	0.35	0.35	0.35	0.35	0.40	0.40	0.40	0.40	0.40	0.40	0.45	0.45	0.45	0.45	0.45
pI	0.06	-0.14	0.06	0.06	0.26	0.06	0.06	-0.14	-0.14	0.26	0.26	-0.14	-0.14	0.06	0.06	0.06	0.06	0.26	0.26	-0.14	-0.14	-0.14	-0.14	0.26	0.26	0.26	0.26	-0.34	0.06	0.06	0.46	0.06	0.06	-0.34	-0.34	-0.14	-0.14	0.26
ps	1.44	1.44	1.44	1.44	1.44	1.24	1.64	1.44	1.44	1.44	1.44	1.24	1.64	1.24	1.24	1.64	1.64	1.24	1.64	1.24	1.24	1.64	1.64	1.24	1.24	1.64	1.64	1.44	1.44	1.44	1.44	1.04	1.84	1.44	1.44	1.44	1.44	1.44
pF	0.13	0.13	-0.07	0.33	0.13	0.13	0.13	-0.07	0.33	-0.07	0.33	0.13	0.13	-0.07	0.33	-0.07	0.33	0.13	0.13	-0.07	0.33	-0.07	0.33	-0.07	0.33	-0.07	0.33	0.13	-0.27	0.53	0.13	0.13	0.13	-0.07	0.33	-0.27	0.53	-0.27

index	39	40	41	42	43	44	45	46	47	48	49	50	51	52	53	54	55	56	57	58	59	60	61	62	63	64	65	66	67	68	69	70	71	72	73	74	75	76
distance	0.45	0.45	0.45	0.45	0.45	0.45	0.45	0.45	0.45	0.45	0.45	0.45	0.45	0.45	0.45	0.45	0.45	0.45	0.45	0.49	0.49	0.49	0.49	0.49	0.49	0.49	0.49	0.49	0.49	0.49	0.49	0.49	0.49	0.49	0.49	0.49	0.49	0.49
pI	0.26	0.46	0.46	-0.34	-0.34	0.06	0.06	0.06	0.06	0.46	0.46	-0.14	-0.14	0.06	0.06	0.06	0.06	0.26	0.26	-0.34	-0.34	-0.34	-0.34	-0.14	-0.14	-0.14	-0.14	0.26	0.26	0.26	0.26	0.46	0.46	0.46	0.46	-0.14	-0.14	-0.14
ps	1.44	1.44	1.44	1.24	1.64	1.24	1.24	1.64	1.64	1.24	1.64	1.04	1.84	1.04	1.04	1.84	1.84	1.04	1.84	1.24	1.24	1.64	1.64	1.24	1.24	1.64	1.64	1.24	1.24	1.64	1.64	1.24	1.24	1.64	1.64	1.04	1.04	1.84
pF	0.53	-0.07	0.33	0.13	0.13	-0.27	0.53	-0.27	0.53	0.13	0.13	0.13	0.13	-0.07	0.33	-0.07	0.33	0.13	0.13	-0.07	0.33	-0.07	0.33	-0.27	0.53	-0.27	0.53	-0.27	0.53	-0.27	0.53	-0.07	0.33	-0.07	0.33	-0.07	0.33	-0.07

index	77	78	79	80	81	82	83	84	85	86	87	88	89	90	91	92	93	94	95	96	97	98	99	100	101	102	103	104	105	106	107	108	109	110	111	112	113	114
distance	0.49	0.49	0.49	0.49	0.49	0.57	0.57	0.57	0.57	0.57	0.57	0.57	0.57	0.57	0.57	0.57	0.57	0.60	0.60	0.60	0.60	0.60	0.60	0.60	0.60	0.60	0.60	0.60	0.60	0.60	0.60	0.60	0.60	0.60	0.60	0.60	0.60	0.60
pI	-0.14	0.26	0.26	0.26	0.26	-0.34	-0.34	-0.34	-0.34	0.06	0.06	0.06	0.06	0.46	0.46	0.46	0.46	-0.34	-0.34	-0.34	-0.34	-0.14	-0.14	-0.14	-0.14	0.26	0.26	0.26	0.26	0.46	0.46	0.46	0.46	-0.34	-0.34	-0.34	-0.34	0.46
ps	1.84	1.04	1.04	1.84	1.84	1.04	1.44	1.44	1.84	1.04	1.04	1.84	1.84	1.04	1.44	1.44	1.84	1.24	1.24	1.64	1.64	1.04	1.04	1.84	1.84	1.04	1.04	1.84	1.84	1.24	1.24	1.64	1.64	1.04	1.04	1.84	1.84	1.04
pF	0.33	-0.07	0.33	-0.07	0.33	0.13	-0.27	0.53	0.13	-0.27	0.53	-0.27	0.53	0.13	-0.27	0.53	0.13	-0.27	0.53	-0.27	0.53	-0.27	0.53	-0.27	0.53	-0.27	0.53	-0.27	0.53	-0.27	0.53	-0.27	0.53	-0.07	0.33	-0.07	0.33	-0.07

index	115	116	117	118	119	120	121	122	123	124	125	126	127	128	129	130	131	132	133	134	135	136	137	138	139	140	141	142	143	144	145	146	147	148	149	150	151	152
distance	0.60	0.60	0.60	0.69	0.69	0.69	0.69	0.69	0.69	0.69	0.69	2.02	2.61	2.64	2.86	3.11	3.31	3.33	3.40	3.44	3.72	3.79	3.80	3.81	3.96	3.99	4.13	4.15	4.31	4.32	4.39	4.46	4.67	4.70	4.84	4.97	4.98	5.44
pI	0.46	0.46	0.46	-0.34	-0.34	-0.34	-0.34	0.46	0.46	0.46	0.46	1.00	1.00	2.00	1.00	2.00	1.00	2.00	1.00	3.00	2.00	1.00	2.00	3.00	1.00	3.00	1.00	2.00	2.00	3.00	3.00	2.00	1.00	3.00	3.00	2.00	3.00	3.00
ps	1.04	1.84	1.84	1.04	1.04	1.84	1.84	1.04	1.04	1.84	1.84	3.00	3.00	3.00	4.00	3.00	4.00	4.00	3.00	3.00	4.00	5.00	3.00	3.00	4.00	4.00	5.00	5.00	4.00	4.00	3.00	5.00	5.00	5.00	4.00	5.00	5.00	5.00
pF	0.33	-0.07	0.33	-0.27	0.53	-0.27	0.53	-0.27	0.53	-0.27	0.53	1.00	2.00	1.00	1.00	2.00	2.00	1.00	3.00	1.00	2.00	1.00	3.00	2.00	3.00	1.00	2.00	1.00	3.00	2.00	3.00	2.00	3.00	1.00	3.00	3.00	2.00	3.00

Deer 3

index	1	2	3	4	5	6	7	8	9	10	11	12	13	14	15	16	17	18	19	20	21	22	23	24	25	26	27	28	29	30	31	32	33	34	35	36	37	38
distance	0.00	0.20	0.20	0.20	0.20	0.20	0.20	0.28	0.28	0.28	0.28	0.28	0.28	0.28	0.28	0.28	0.28	0.28	0.28	0.35	0.35	0.35	0.35	0.35	0.35	0.35	0.35	0.40	0.40	0.40	0.40	0.40	0.40	0.45	0.45	0.45	0.45	0.45
pI	0.12	-0.08	0.12	0.12	0.32	0.12	0.12	-0.08	-0.08	0.32	0.32	-0.08	-0.08	0.12	0.12	0.12	0.12	0.32	0.32	-0.08	-0.08	-0.08	-0.08	0.32	0.32	0.32	0.32	-0.28	0.12	0.12	0.52	0.12	0.12	-0.28	-0.28	-0.08	-0.08	0.32
ps	1.70	1.70	1.70	1.70	1.70	1.50	1.90	1.70	1.70	1.70	1.70	1.50	1.90	1.50	1.50	1.90	1.90	1.50	1.90	1.50	1.50	1.90	1.90	1.50	1.50	1.90	1.90	1.70	1.70	1.70	1.70	1.30	2.10	1.70	1.70	1.70	1.70	1.70
pF	0.05	0.05	-0.15	0.25	0.05	0.05	0.05	-0.15	0.25	-0.15	0.25	0.05	0.05	-0.15	0.25	-0.15	0.25	0.05	0.05	-0.15	0.25	-0.15	0.25	-0.15	0.25	-0.15	0.25	0.05	-0.35	0.45	0.05	0.05	0.05	-0.15	0.25	-0.35	0.45	-0.35

index	39	40	41	42	43	44	45	46	47	48	49	50	51	52	53	54	55	56	57	58	59	60	61	62	63	64	65	66	67	68	69	70	71	72	73	74	75	76
distance	0.45	0.45	0.45	0.45	0.45	0.45	0.45	0.45	0.45	0.45	0.45	0.45	0.45	0.45	0.45	0.45	0.45	0.45	0.45	0.49	0.49	0.49	0.49	0.49	0.49	0.49	0.49	0.49	0.49	0.49	0.49	0.49	0.49	0.49	0.49	0.49	0.49	0.49
pI	0.32	0.52	0.52	-0.28	-0.28	0.12	0.12	0.12	0.12	0.52	0.52	-0.08	-0.08	0.12	0.12	0.12	0.12	0.32	0.32	-0.28	-0.28	-0.28	-0.28	-0.08	-0.08	-0.08	-0.08	0.32	0.32	0.32	0.32	0.52	0.52	0.52	0.52	-0.08	-0.08	-0.08
ps	1.70	1.70	1.70	1.50	1.90	1.50	1.50	1.90	1.90	1.50	1.90	1.30	2.10	1.30	1.30	2.10	2.10	1.30	2.10	1.50	1.50	1.90	1.90	1.50	1.50	1.90	1.90	1.50	1.50	1.90	1.90	1.50	1.50	1.90	1.90	1.30	1.30	2.10
pF	0.45	-0.15	0.25	0.05	0.05	-0.35	0.45	-0.35	0.45	0.05	0.05	0.05	0.05	-0.15	0.25	-0.15	0.25	0.05	0.05	-0.15	0.25	-0.15	0.25	-0.35	0.45	-0.35	0.45	-0.35	0.45	-0.35	0.45	-0.15	0.25	-0.15	0.25	-0.15	0.25	-0.15

index	77	78	79	80	81	82	83	84	85	86	87	88	89	90	91	92	93	94	95	96	97	98	99	100	101	102	103	104	105	106	107	108	109	110	111	112	113	114
distance	0.49	0.49	0.49	0.49	0.49	0.57	0.57	0.57	0.57	0.57	0.57	0.57	0.57	0.57	0.57	0.57	0.57	0.60	0.60	0.60	0.60	0.60	0.60	0.60	0.60	0.60	0.60	0.60	0.60	0.60	0.60	0.60	0.60	0.60	0.60	0.60	0.60	0.60
pI	-0.08	0.32	0.32	0.32	0.32	-0.28	-0.28	-0.28	-0.28	0.12	0.12	0.12	0.12	0.52	0.52	0.52	0.52	-0.28	-0.28	-0.28	-0.28	-0.08	-0.08	-0.08	-0.08	0.32	0.32	0.32	0.32	0.52	0.52	0.52	0.52	-0.28	-0.28	-0.28	-0.28	0.52
ps	2.10	1.30	1.30	2.10	2.10	1.30	1.70	1.70	2.10	1.30	1.30	2.10	2.10	1.30	1.70	1.70	2.10	1.50	1.50	1.90	1.90	1.30	1.30	2.10	2.10	1.30	1.30	2.10	2.10	1.50	1.50	1.90	1.90	1.30	1.30	2.10	2.10	1.30
pF	0.25	-0.15	0.25	-0.15	0.25	0.05	-0.35	0.45	0.05	-0.35	0.45	-0.35	0.45	0.05	-0.35	0.45	0.05	-0.35	0.45	-0.35	0.45	-0.35	0.45	-0.35	0.45	-0.35	0.45	-0.35	0.45	-0.35	0.45	-0.35	0.45	-0.15	0.25	-0.15	0.25	-0.15

index	115	116	117	118	119	120	121	122	123	124	125	126	127	128	129	130	131	132	133	134	135	136	137	138	139	140	141	142	143	144	145	146	147	148	149	150	151	152
distance	0.60	0.60	0.60	0.69	0.69	0.69	0.69	0.69	0.69	0.69	0.69	1.84	2.48	2.50	2.64	3.01	3.12	3.14	3.30	3.34	3.55	3.56	3.71	3.73	3.81	3.84	3.92	3.93	4.17	4.19	4.27	4.32	4.49	4.51	4.72	4.80	4.81	5.28
pI	0.52	0.52	0.52	-0.28	-0.28	-0.28	-0.28	0.52	0.52	0.52	0.52	1.00	2.00	1.00	1.00	2.00	2.00	1.00	3.00	1.00	1.00	2.00	3.00	2.00	3.00	1.00	2.00	1.00	3.00	2.00	2.00	3.00	3.00	1.00	3.00	3.00	2.00	3.00
ps	1.30	2.10	2.10	1.30	1.30	2.10	2.10	1.30	1.30	2.10	2.10	3.00	3.00	3.00	4.00	3.00	4.00	4.00	3.00	3.00	5.00	4.00	3.00	3.00	4.00	4.00	5.00	5.00	4.00	4.00	5.00	3.00	5.00	5.00	4.00	5.00	5.00	5.00
pF	0.25	-0.15	0.25	-0.35	0.45	-0.35	0.45	-0.35	0.45	-0.35	0.45	1.00	1.00	2.00	1.00	2.00	1.00	2.00	1.00	3.00	1.00	2.00	2.00	3.00	1.00	3.00	1.00	2.00	2.00	3.00	2.00	3.00	1.00	3.00	3.00	2.00	3.00	3.00

Deer 4

Label	1	2	3	4	5	6	7	8	9	10	11	12	13	14	15	16	17	18	19	20	21	22	23	24	25	26	27	28	29	30	31	32	33	34	35	36	37	38
distance	0.00	0.20	0.20	0.20	0.20	0.20	0.20	0.28	0.28	0.28	0.28	0.28	0.28	0.28	0.28	0.28	0.28	0.28	0.28	0.35	0.35	0.35	0.35	0.35	0.35	0.35	0.35	0.40	0.40	0.40	0.40	0.40	0.40	0.45	0.45	0.45	0.45	0.45
pI	0.10	-0.10	0.10	0.10	0.30	0.10	0.10	-0.10	-0.10	0.30	0.30	-0.10	-0.10	0.10	0.10	0.10	0.10	0.30	0.30	-0.10	-0.10	-0.10	-0.10	0.30	0.30	0.30	0.30	-0.30	0.10	0.10	0.50	0.10	0.10	-0.30	-0.30	-0.10	-0.10	0.30
ps	1.52	1.52	1.52	1.52	1.52	1.32	1.72	1.52	1.52	1.52	1.52	1.32	1.72	1.32	1.32	1.72	1.72	1.32	1.72	1.32	1.32	1.72	1.72	1.32	1.32	1.72	1.72	1.52	1.52	1.52	1.52	1.12	1.92	1.52	1.52	1.52	1.52	1.52
pF	0.06	0.06	-0.14	0.26	0.06	0.06	0.06	-0.14	0.26	-0.14	0.26	0.06	0.06	-0.14	0.26	-0.14	0.26	0.06	0.06	-0.14	0.26	-0.14	0.26	-0.14	0.26	-0.14	0.26	0.06	-0.34	0.46	0.06	0.06	0.06	-0.14	0.26	-0.34	0.46	-0.34

Label	39	40	41	42	43	44	45	46	47	48	49	50	51	52	53	54	55	56	57	58	59	60	61	62	63	64	65	66	67	68	69	70	71	72	73	74	75	76
distance	0.45	0.45	0.45	0.45	0.45	0.45	0.45	0.45	0.45	0.45	0.45	0.45	0.45	0.45	0.45	0.45	0.45	0.45	0.45	0.49	0.49	0.49	0.49	0.49	0.49	0.49	0.49	0.49	0.49	0.49	0.49	0.49	0.49	0.49	0.49	0.49	0.49	0.49
pI	0.30	0.50	0.50	-0.30	-0.30	0.10	0.10	0.10	0.10	0.50	0.50	-0.10	-0.10	0.10	0.10	0.10	0.10	0.30	0.30	-0.30	-0.30	-0.30	-0.30	-0.10	-0.10	-0.10	-0.10	0.30	0.30	0.30	0.30	0.50	0.50	0.50	0.50	-0.10	-0.10	-0.10
ps	1.52	1.52	1.52	1.32	1.72	1.32	1.32	1.72	1.72	1.32	1.72	1.12	1.92	1.12	1.12	1.92	1.92	1.12	1.92	1.32	1.32	1.72	1.72	1.32	1.32	1.72	1.72	1.32	1.32	1.72	1.72	1.32	1.32	1.72	1.72	1.12	1.12	1.92
pF	0.46	-0.14	0.26	0.06	0.06	-0.34	0.46	-0.34	0.46	0.06	0.06	0.06	0.06	-0.14	0.26	-0.14	0.26	0.06	0.06	-0.14	0.26	-0.14	0.26	-0.34	0.46	-0.34	0.46	-0.34	0.46	-0.34	0.46	-0.14	0.26	-0.14	0.26	-0.14	0.26	-0.14

Label	77	78	79	80	81	82	83	84	85	86	87	88	89	90	91	92	93	94	95	96	97	98	99	100	101	102	103	104	105	106	107	108	109	110	111	112	113	114
distance	0.49	0.49	0.49	0.49	0.49	0.57	0.57	0.57	0.57	0.57	0.57	0.57	0.57	0.57	0.57	0.57	0.57	0.60	0.60	0.60	0.60	0.60	0.60	0.60	0.60	0.60	0.60	0.60	0.60	0.60	0.60	0.60	0.60	0.60	0.60	0.60	0.60	0.60
pI	-0.10	0.30	0.30	0.30	0.30	-0.30	-0.30	-0.30	-0.30	0.10	0.10	0.10	0.10	0.50	0.50	0.50	0.50	-0.30	-0.30	-0.30	-0.30	-0.10	-0.10	-0.10	-0.10	0.30	0.30	0.30	0.30	0.50	0.50	0.50	0.50	-0.30	-0.30	-0.30	-0.30	0.50
ps	1.92	1.12	1.12	1.92	1.92	1.12	1.52	1.52	1.92	1.12	1.12	1.92	1.92	1.12	1.52	1.52	1.92	1.32	1.32	1.72	1.72	1.12	1.12	1.92	1.92	1.12	1.12	1.92	1.92	1.32	1.32	1.72	1.72	1.12	1.12	1.92	1.92	1.12
pF	0.26	-0.14	0.26	-0.14	0.26	0.06	-0.34	0.46	0.06	-0.34	0.46	-0.34	0.46	0.06	-0.34	0.46	0.06	-0.34	0.46	-0.34	0.46	-0.34	0.46	-0.34	0.46	-0.34	0.46	-0.34	0.46	-0.34	0.46	-0.34	0.46	-0.14	0.26	-0.14	0.26	-0.14

Label	115	116	117	118	119	120	121	122	123	124	125	126	127	128	129	130	131	132	133	134	135	136	137	138	139	140	141	142	143	144	145	146	147	148	149	150	151	152
distance	0.60	0.60	0.60	0.69	0.69	0.69	0.69	0.69	0.69	0.69	0.69	1.97	2.59	2.60	2.80	3.09	3.26	3.28	3.39	3.41	3.68	3.72	3.79	3.80	3.93	3.95	4.08	4.09	4.28	4.29	4.39	4.42	4.63	4.64	4.82	4.93	4.94	5.40
pI	0.50	0.50	0.50	-0.30	-0.30	-0.30	-0.30	0.50	0.50	0.50	0.50	1.00	2.00	1.00	1.00	2.00	2.00	1.00	3.00	1.00	2.00	1.00	3.00	2.00	3.00	1.00	2.00	1.00	3.00	2.00	3.00	2.00	3.00	1.00	3.00	3.00	2.00	3.00
ps	1.12	1.92	1.92	1.12	1.12	1.92	1.92	1.12	1.12	1.92	1.92	3.00	3.00	3.00	4.00	3.00	4.00	4.00	3.00	3.00	4.00	5.00	3.00	3.00	4.00	4.00	5.00	5.00	4.00	4.00	3.00	5.00	5.00	5.00	4.00	5.00	5.00	5.00
pF	0.26	-0.14	0.26	-0.34	0.46	-0.34	0.46	-0.34	0.46	-0.34	0.46	1.00	1.00	2.00	1.00	2.00	1.00	2.00	1.00	3.00	2.00	1.00	2.00	3.00	1.00	3.00	1.00	2.00	2.00	3.00	3.00	2.00	1.00	3.00	3.00	2.00	3.00	3.00

Deer 5

Label	1	2	3	4	5	6	7	8	9	10	11	12	13	14	15	16	17	18	19	20	21	22	23	24	25	26	27	28	29	30	31	32	33	34	35	36	37	38
distance	0.00	0.20	0.20	0.20	0.20	0.20	0.20	0.28	0.28	0.28	0.28	0.28	0.28	0.28	0.28	0.28	0.28	0.28	0.28	0.35	0.35	0.35	0.35	0.35	0.35	0.35	0.35	0.40	0.40	0.40	0.40	0.40	0.40	0.45	0.45	0.45	0.45	0.45
pI	0.22	0.02	0.22	0.22	0.42	0.22	0.22	0.02	0.02	0.42	0.42	0.02	0.02	0.22	0.22	0.22	0.22	0.42	0.42	0.02	0.02	0.02	0.02	0.42	0.42	0.42	0.42	-0.18	0.22	0.22	0.62	0.22	0.22	-0.18	-0.18	0.02	0.02	0.42
ps	1.66	1.66	1.66	1.66	1.66	1.46	1.86	1.66	1.66	1.66	1.66	1.46	1.86	1.46	1.46	1.86	1.86	1.46	1.86	1.46	1.46	1.86	1.86	1.46	1.46	1.86	1.86	1.66	1.66	1.66	1.66	1.26	2.06	1.66	1.66	1.66	1.66	1.66
pF	0.24	0.24	0.04	0.44	0.24	0.24	0.24	0.04	0.44	0.04	0.44	0.24	0.24	0.04	0.44	0.04	0.44	0.24	0.24	0.04	0.44	0.04	0.44	0.04	0.44	0.04	0.44	0.24	-0.16	0.64	0.24	0.24	0.24	0.04	0.44	-0.16	0.64	-0.16

Label	39	40	41	42	43	44	45	46	47	48	49	50	51	52	53	54	55	56	57	58	59	60	61	62	63	64	65	66	67	68	69	70	71	72	73	74	75	76
distance	0.45	0.45	0.45	0.45	0.45	0.45	0.45	0.45	0.45	0.45	0.45	0.45	0.45	0.45	0.45	0.45	0.45	0.45	0.45	0.49	0.49	0.49	0.49	0.49	0.49	0.49	0.49	0.49	0.49	0.49	0.49	0.49	0.49	0.49	0.49	0.49	0.49	0.49
pI	0.42	0.62	0.62	-0.18	-0.18	0.22	0.22	0.22	0.22	0.62	0.62	0.02	0.02	0.22	0.22	0.22	0.22	0.42	0.42	-0.18	-0.18	-0.18	-0.18	0.02	0.02	0.02	0.02	0.42	0.42	0.42	0.42	0.62	0.62	0.62	0.62	0.02	0.02	0.02
ps	1.66	1.66	1.66	1.46	1.86	1.46	1.46	1.86	1.86	1.46	1.86	1.26	2.06	1.26	1.26	2.06	2.06	1.26	2.06	1.46	1.46	1.86	1.86	1.46	1.46	1.86	1.86	1.46	1.46	1.86	1.86	1.46	1.46	1.86	1.86	1.26	1.26	2.06
pF	0.64	0.04	0.44	0.24	0.24	-0.16	0.64	-0.16	0.64	0.24	0.24	0.24	0.24	0.04	0.44	0.04	0.44	0.24	0.24	0.04	0.44	0.04	0.44	-0.16	0.64	-0.16	0.64	-0.16	0.64	-0.16	0.64	0.04	0.44	0.04	0.44	0.04	0.44	0.04

Label	77	78	79	80	81	82	83	84	85	86	87	88	89	90	91	92	93	94	95	96	97	98	99	100	101	102	103	104	105	106	107	108	109	110	111	112	113	114
distance	0.49	0.49	0.49	0.49	0.49	0.57	0.57	0.57	0.57	0.57	0.57	0.57	0.57	0.57	0.57	0.57	0.57	0.60	0.60	0.60	0.60	0.60	0.60	0.60	0.60	0.60	0.60	0.60	0.60	0.60	0.60	0.60	0.60	0.60	0.60	0.60	0.60	0.60
pI	0.02	0.42	0.42	0.42	0.42	-0.18	-0.18	-0.18	-0.18	0.22	0.22	0.22	0.22	0.62	0.62	0.62	0.62	-0.18	-0.18	-0.18	-0.18	0.02	0.02	0.02	0.02	0.42	0.42	0.42	0.42	0.62	0.62	0.62	0.62	-0.18	-0.18	-0.18	-0.18	0.62
ps	2.06	1.26	1.26	2.06	2.06	1.26	1.66	1.66	2.06	1.26	1.26	2.06	2.06	1.26	1.66	1.66	2.06	1.46	1.46	1.86	1.86	1.26	1.26	2.06	2.06	1.26	1.26	2.06	2.06	1.46	1.46	1.86	1.86	1.26	1.26	2.06	2.06	1.26
pF	0.44	0.04	0.44	0.04	0.44	0.24	-0.16	0.64	0.24	-0.16	0.64	-0.16	0.64	0.24	-0.16	0.64	0.24	-0.16	0.64	-0.16	0.64	-0.16	0.64	-0.16	0.64	-0.16	0.64	-0.16	0.64	-0.16	0.64	-0.16	0.64	0.04	0.44	0.04	0.44	0.04

Label	115	116	117	118	119	120	121	122	123	124	125	126	127	128	129	130	131	132	133	134	135	136	137	138	139	140	141	142	143	144	145	146	147	148	149	150	151	152
distance	0.60	0.60	0.60	0.69	0.69	0.69	0.69	0.69	0.69	0.69	0.69	1.73	2.35	2.36	2.58	2.84	3.03	3.04	3.17	3.18	3.43	3.51	3.55	3.56	3.70	3.71	3.86	3.86	4.03	4.04	4.14	4.18	4.40	4.41	4.57	4.69	4.69	5.15
pI	0.62	0.62	0.62	-0.18	-0.18	-0.18	-0.18	0.62	0.62	0.62	0.62	1.00	1.00	2.00	1.00	2.00	1.00	2.00	1.00	3.00	2.00	1.00	2.00	3.00	1.00	3.00	1.00	2.00	2.00	3.00	3.00	2.00	1.00	3.00	3.00	2.00	3.00	3.00
ps	1.26	2.06	2.06	1.26	1.26	2.06	2.06	1.26	1.26	2.06	2.06	3.00	3.00	3.00	4.00	3.00	4.00	4.00	3.00	3.00	4.00	5.00	3.00	3.00	4.00	4.00	5.00	5.00	4.00	4.00	3.00	5.00	5.00	5.00	4.00	5.00	5.00	5.00
pF	0.44	0.04	0.44	-0.16	0.64	-0.16	0.64	-0.16	0.64	-0.16	0.64	1.00	2.00	1.00	1.00	2.00	2.00	1.00	3.00	1.00	2.00	1.00	3.00	2.00	3.00	1.00	2.00	1.00	3.00	2.00	3.00	2.00	3.00	1.00	3.00	3.00	2.00	3.00
