# Supplementary material for: A random walk model that accounts for space occupation and movements of a large herbivore
Source: Sci Rep. 2021 Jul 7;11:14061. doi: 10.1038/s41598-021-93387-2 (PMC8263821; doi:10.1038/s41598-021-93387-2)

A random walk model that accounts for space occupation and movements of a large herbivore

Geoffroy Berthelot[1,2,3], Sonia Saïd[4], and Vincent Bansaye[1]
1 Ecole Polytechnique, Centre de mathématiques appliquées (CMAP), Palaiseau, 91128, France
2 REsearch LAboratory for Interdisciplinary Studies (RELAIS), Paris, 75012, France
3 Institut national du sport, de l'expertise et de la performance (INSEP), Paris, 75012, France
4 Office Français de la Biodiversité, Direction Recherche et Appui Scientifique, Unité Ongulés Sauvages-Unité Flore et Végétation, Birieux, 01330, France

Supplementary Figure S6

The alpha shape using a fixed alpha radius of 60m are presented for all deers (green areas). Only the localizations near the isobarycenter are displayed here and distant localizations are not showed. Voids of interest are near the center of the shapes, and labelled with circled numbers.

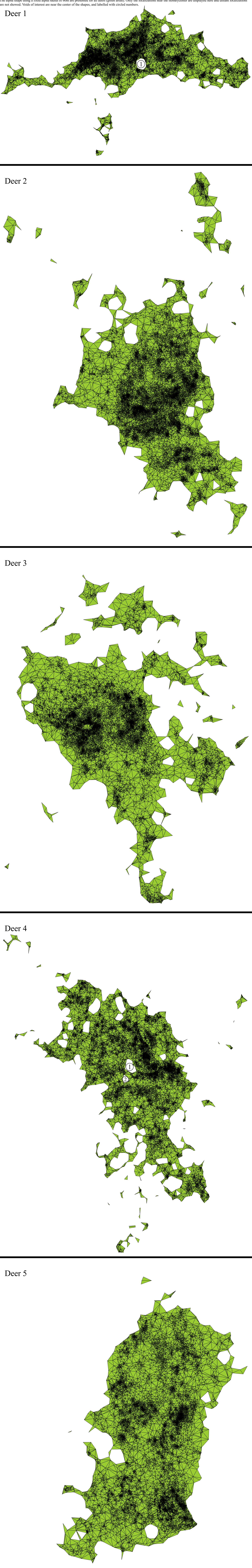

Supplement: Supplementary file 6 — Supplementary Figure 6 [file 41598_2021_93387_MOESM6_ESM.pdf]
