# Supplementary material for: A random walk model that accounts for space occupation and movements of a large herbivore
Source: Sci Rep. 2021 Jul 7;11:14061. doi: 10.1038/s41598-021-93387-2 (PMC8263821; doi:10.1038/s41598-021-93387-2)
Supplement: Supplementary file 7 — Supplementary Methods [file 41598_2021_93387_MOESM7_ESM.pdf]

A random walk model that accounts for space occupation and movements of a large herbivore
Geoffroy Berthelot[1,2,3], Sonia Saïd[4], and Vincent Bansaye[1]
[1] Ecole Polytechnique, Centre de mathématiques appliquées (CMAP), Palaiseau, 91128, France
[2] REsearch LAboratory for Interdisciplinary Studies (RELAIS), Paris, 75012, France
[3] Institut national du sport, de l'expertise et de la performance (INSEP), Paris, 75012, France
[4] Office Français de la Biodiversité, Direction Recherche et Appui Scientifique, Unité Ongulés Sauvages-Unité Flore et Végétation, Birieux, 01330, France

Supplementary Methods

We here provide additional information regarding some technical aspects of the statistics.

1 Empirical estimation of the parameters

The estimation of the three parameters was achieved through a spatial discretization. For each time step i, we discretized the space around the animal in 8 quadrants. This can be represented as a 3×3 matrix M corresponding to the motion of the animal at each time step with a particular arrangement of the three parameters, depending on both the animal and den position, and the previous motion. For illustration, the change from spatial quadrants to a matrix representation can be pictured as follow:

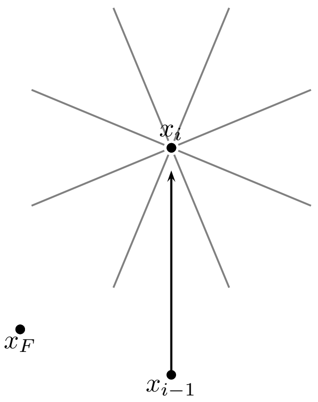

$$M_i = \begin{bmatrix} 1 & 1+p_I & 1 \\ 1 & p_s & 1 \\ 1+p_F & 1 & 1 \end{bmatrix} \quad (19)$$

with the graphic element (left) representing the animal state from step $i-1$ to step i. The matrix M_i (eq. 19) is the associated matrix describing this state. The central element in position $(2,2)$ corresponds to the animal (particle) position x_i. The space around the animal was divided in 8 quadrants and the positions of p_I and p_F in the matrix changed at each time step, depending on the previous direction (parameter p_I) and the position of the den (parameter p_F). In the presented case (eq. 19), the animal is heading north (*i.e.* upward vertical direction $\uparrow$ from x_{i-1}) such that the parameter p_I is located in the matrix at position $(1,2)$. The parameter p_F is located at position $(3,1)$ because the den is located in the third quadrant respectively to the position of the animal.

The matrix M (eq. 19), normalized by $8 + p_I + p_s + p_F$, is the matrix representation of the transition rates from a given state to itself and to its 8 nearest and next to nearest neighbours. Using this method, we were able to collect enough observations for estimating each parameter, provided the states are in $\mathcal{H}$ (eq. 6). We did not evaluate the combination of the parameters for the conflicting states $\mathcal{H}_{IF}$ and $\mathcal{H}_{Is}$. Instead, after estimating the parameters and during the simulation of the BCR model, we combined the parameters when conflicting states $\mathcal{H}_{IF}$ and $\mathcal{H}_{Is}$ occur as follow:

$$M_i = \begin{bmatrix} 1 & 1+p_I+p_F & 1 \\ 1 & p_s & 1 \\ 1 & 1 & 1 \end{bmatrix}$$

when the individual is performing consecutive steps toward the den (ie. in $\mathcal{H}_{IF}$) and

$$M_i = \begin{bmatrix} 1 & 1 & 1 \\ 1 & p_s + p_I & 1 \\ 1 + p_F & 1 & 1 \end{bmatrix}$$

when the individual is considered immobile for successive steps (ie. in $\mathcal{H}_{Is}$).

2 Negative parameters

It is possible to obtain negative parameters when using the method above. It may be related to a flaw in the design of the BCR such as ignoring forces that have a significant effect on the movement. It can also be viewed as a repellent parameter, when the animal avoid a specific direction. Using the matrix representation (eq. 19), and considering a single negative parameter $-1 \leq p_n < 0$, it is possible to simulate a "short-term avoiding" walk using the following modification:

$$p = \frac{7 - p_n}{8}$$

with p the probability to avoid the desired direction, *i.e.* to realize the movement in the remaining 7 spatial quadrants (eq. 19), and:

$$1 - p = \frac{1 + p_n}{8}$$

the probability of the opposite event, *i.e* to go to the unwanted direction. For instance, considering the model with inertia only and setting $p_I = -1$ will result in a movement with no inertia at all. It means the animal movement will never perform inertia thus always favoring other directions.

3 Distribution of turning angles

We focused on the normalized histogram m_j containing $j = 1, \ldots, 20$ bins in the $[-\pi, \pi]$ interval. The function m counts the number of angular values that fall into each of the bins. The normalized histogram m_j for the empirical dataset meets the following condition:

$$\sum_{j=1}^{20} m_j = 1$$

and the normalized histogram $\hat{m}_{j,k}$ for a given realization k of the BCR is:

$$\sum_{j=1}^{20} \hat{m}_{j,k} = 1$$

This normalization remove the effect of immobilism p_s as m_j is only based on non-immobile ($d > d_{\min}$) observations. We rather focused on the shape of the distribution than on the absolute number of values in each bin. For one BCR realization k, the differences between the data and the simulated paths in each of the 20 bins were computed as:

$$e_{1_k} = \sum_{j=1}^{20} \left| m_j - \hat{m}_{j,k} \right|$$

For error e_1. And for error e_2 we used:

$$e_{2_k} = \frac{1}{20} \sum_{j=1}^{20} \frac{\hat{m}_{j,k}}{m_j}$$

such that error e_2 for this statistic and one given realization k is the average of the relative errors between the each bins.

4 Transects

In the transects statistic, we used two main types of transects: immobile transects and mobile transects. Immobile transects are fixed transects that count the number of time the animal appears in their line of sight. Mobile transects are similar, except they move according to a predefined pattern. Each transect is characterized by a line of sight and a speed (for mobile transects) and we studied several line of sights and speeds (see methodological section in the main article). For each value of line of sight and speed, we ran a statistic using the (empirical or simulated) animal path and the (immobile or mobiles) transects, starting at time t_1. We stopped the statistic when reaching t_n and simply summed the count for all transects. It means that each value of line of sight and speed contains the sum of observations of the animal by the transects.

4.1 Immobile transects

A mesh m is defined with r nodes that encapsulate all the locations X_i of the animal (supporting Fig. 2 and graphic 1):

$$\begin{aligned} x_0 &= \min\left(X_i^{(1)}\right) \\ y_0 &= \min\left(X_i^{(2)}\right) \\ x_M &= \max\left(X_i^{(1)}\right) \\ y_M &= \max\left(X_i^{(2)}\right) \end{aligned} \tag{20}$$

$[x_0, y_0]$ and $[x_M, y_M]$ being the boundaries of m with a constant spacing value a between each node. Transects counts were then gathered and ordered in increasing order. We previously compared several

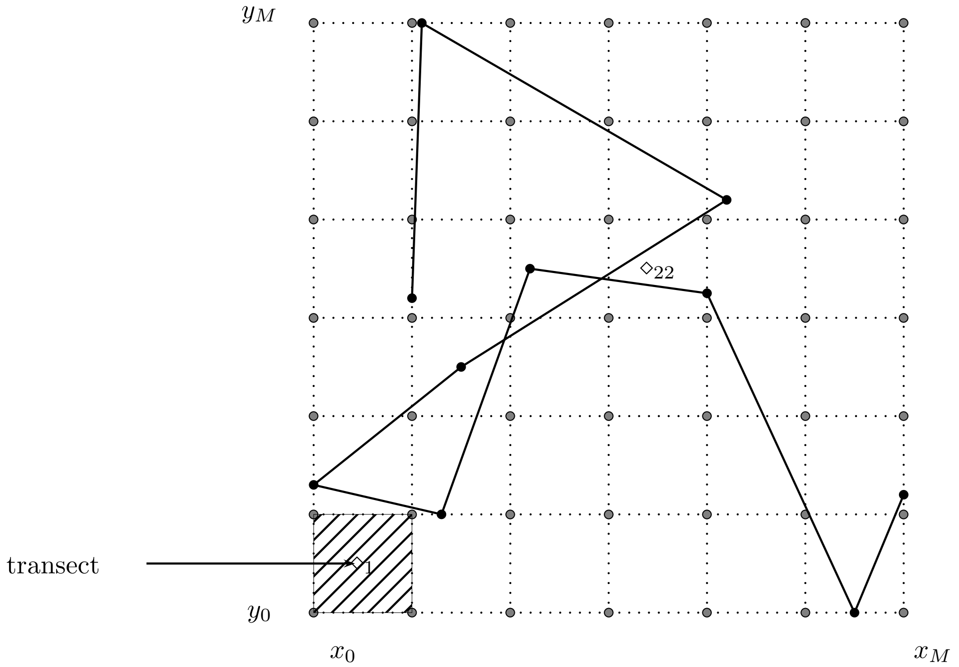

Graphic 1 – A 7×7 mesh ($r = 49$ nodes) of 36 immobile transects. Each cell of the mesh corresponds to an observation area of one immobile transect. The shaded area of the lower left cell corresponds to the area of vision of the transect $\diamond_1$. Transects are ordered in a column zig-zag way. The path (black line and dots) is counted in each cell, such that the value of $\diamond_1$ is 0 and $\diamond_{22} = 2$.

meshes with different resolutions (i.e. different values of a, r) and it only impacted the results by a scale factor.

4.2 Mobile transects

The issue was to find the location of both the animal and the mobile transects at each time step.

4.2.1 Location of the animal

The path of the animal was reconstructed from the locations X_i. Linear interpolation was used between each pair of recorded locations, as detailed in Lonergan et al. (2009). It assumes the animal travels in straight lines at constant velocity between each pair of locations. Such that the path of the animal is a piecewise linear function of the observations:

$$X_i^{(2)} = \begin{cases} \alpha_1 \cdot X_1^{(1)} + \beta_1 & \text{if } t \leq t_{X_2} \\ \alpha_2 \cdot X_2^{(1)} + \beta_2 & \text{if } t_{X_1} < t \leq t_{X_3} \\ \quad \cdots & \\ \alpha_n \cdot X_n^{(1)} + \beta_n & \text{if } t_{X_{n-1}} < t \leq t_{X_n} \end{cases} \tag{21}$$

and for any time t we can find the corresponding observation X_i and define the segment $[X_i, X_{i+1}]$ where the animal is located (graph. 2). Thus the corresponding location of the animal is:

$$X_t = \lambda X_{i+1} + (1 - \lambda) X_i \tag{22}$$

with:

$$\lambda = \frac{t - t_{X_i}}{t_{X_{i+1}} - t_{X_i}} \tag{23}$$

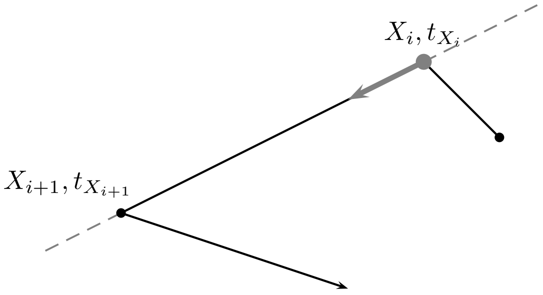

Graphic 2 – The path of an animal considered as a piecewise linear movement.

4.2.2 Location of the mobile linear transect

Let s be the speed of transects and δ the time step. The path of a linear transect with time is:

$$X_{t+\delta} = \begin{cases} X_{t+\delta}^{(1)} = X_t^{(1)} \pm q \cdot s \cdot \delta \\ X_{t+\delta}^{(2)} = X_t^{(2)} \pm q \cdot s \cdot \delta \end{cases} \tag{24}$$

where $q = \{0, 1\}$ depending on vertical or horizontal paths.

4.2.3 Location of the rotating transects

The path of a clockwise rotating transect with time is defined by:

$$X_{t+\delta} = \begin{cases} X_{t+\delta}^{(1)} = -r \cos\left(\alpha_t + \dfrac{s \cdot \delta}{r}\right) + c \\ X_{t+\delta}^{(2)} = -r \sin\left(\alpha_t + \dfrac{s \cdot \delta}{r}\right) + c \end{cases} \tag{25}$$

with radius r, center c and $\left(\alpha_t + \dfrac{s \cdot \delta}{r}\right) = 0$ at t_0.

4.3 Error estimation

In order to compute both errors e_1 and e_2, we aggregated each transects count, meaning that we summed the counts for each lines of sight and speeds and arranged them in a matrix M:

$$M = \begin{bmatrix} c_{11} & c_{12} & c_{13} & c_{14} \\ c_{21} & c_{22} & c_{23} & c_{24} \\ \cdots & \cdots & \cdots & \cdots \\ c_{61} & c_{62} & c_{63} & c_{64} \end{bmatrix}$$

with $c_{i,j}$ the aggregated count for line of sight i and speed j. We then computed e_1 as:

$$e_{1_k} = \left| \sum_{ij} M_{ij} - \sum_{ij} \hat{M}_{kij} \right|$$

where $\hat{M}_k$ is the matrix for BCR realization k. And for error e_2 we used:

$$e_{2_k} = \frac{\sum_{ij} \hat{M}_{kij}}{\sum_{ij} M_{ij}}$$

We repeated the procedure for the rotating transects and computed both errors e_1 and e_2 in the same way.

5 Fluctuations

We investigate the fluctuations of the statistics over a range of increasing n_s values with $n_s = 10^4, 2 \times 10^4, \ldots, 4 \times 10^5$. For each of those step values, a set of 100 BCR is simulated with parameters p_I, p_F and p_s estimated from the first deer (see Table 2). The following quantities are investigated:

1. the intra-bins variance in the normalized histograms of turning angles. For each n_s value, the variance in each of the 20 bins is estimated and we use $\sum_1^{20} \mathrm{Var}(j)$ as an indicator of the overall intra-bins variance, with $\mathrm{Var}(j)$ being the variance of bin j.
2. the intra-density of the normalized home range area. Similarly to 1., we compute the variance of the estimated home range area for each density and for each n_s value. Again, we sum the variances as an indicator of the overall variance as n_s change. The home-range distributions are normalized such as the sum of their densities equals to 1.
3. the variance of the normalized distribution of the first 500 immobile transects counts.
4. the variance of the normalized count in mobile linear and in rotational transects. In order to assess the changes in counts as n_s increase, we compute the variance of the overall counts for the different speeds and radiuses tested. Counts are normalized by the sum of the counts computed for all 100 simulations in every speed and radius.
5. the intra-disk size variance of the dilated paths. Similarly to 1. and for each n_s value, we compute the variance of the estimated surface for each disk size. Again, we use proportions rather than absolute surfaces values: each surface is normalized by the sum of all the surfaces obtained after dilating with all different disk sizes. We sum the variances as an indicator of the overall variance as n_s change.

References

Lonergan M, Fedak M, & McConnell B (2009) The effects of interpolation error and location quality on animal track reconstruction. Marine Mammal Science 25(2):275–282
