# Supplementary material for: A random walk model that accounts for space occupation and movements of a large herbivore
Source: Sci Rep. 2021 Jul 7;11:14061. doi: 10.1038/s41598-021-93387-2 (PMC8263821; doi:10.1038/s41598-021-93387-2)
Supplement: Supplementary file 8 — Supplementary Table 1 [file 41598_2021_93387_MOESM8_ESM.pdf]

A random walk model that accounts for space occupation and movements of a large herbivore

Geoffroy Berthelot[1,2,3], Sonia Saïd[4], and Vincent Bansaye[1]
1 Ecole Polytechnique, Centre de mathématiques appliquées (CMAP), Palaiseau, 91128, France
2 REsearch LAboratory for Interdisciplinary Studies (RELAIS), Paris, 75012, France
3 Institut national du sport, de l'expertise et de la performance (INSEP), Paris, 75012, France
4 Office Français de la Biodiversité, Direction Recherche et Appui Scientifique, Unité Ongulés Sauvages-Unité Flore et Végétation, Birieux, 01330, France

Supplementary Table S1

Parameters values when using only one parameter instead of the three.

**Parameters configuration**	**Deer**	p_I	p_s	p_F
p_I	Deer#1	-0.1742		
p_I	Deer#2	-0.1176		
p_I	Deer#3	-0.0928		
p_I	Deer#4	-0.0774		
p_I	Deer#5	-0.0402		
p_s	Deer#1		1.9718	
p_s	Deer#2		1.3956	
p_s	Deer#3		1.6534	
p_s	Deer#4		1.4871	
p_s	Deer#5		1.6207	
p_F	Deer#1			-0.1746
p_F	Deer#2			-0.0694
p_F	Deer#3			-0.1474
p_F	Deer#4			-0.11
p_F	Deer#5			-0.0164
p_I, p_s, p_F	Deer#1	0.0111	2.0073	0.0121
p_I, p_s, p_F	Deer#2	0.0643	1.4367	0.1316
p_I, p_s, p_F	Deer#3	0.1174	1.697	0.0531
p_I, p_s, p_F	Deer#4	0.101	1.5157	0.063
p_I, p_s, p_F	Deer#5	0.2176	1.6606	0.2382
