# Supplementary material for: A random walk model that accounts for space occupation and movements of a large herbivore
Source: Sci Rep. 2021 Jul 7;11:14061. doi: 10.1038/s41598-021-93387-2 (PMC8263821; doi:10.1038/s41598-021-93387-2)
Supplement: Supplementary file 9 — Supplementary Table 2 [file 41598_2021_93387_MOESM9_ESM.pdf]

**A random walk model that accounts for space occupation and movements of a large herbivore**

Geoffroy Berthelot[1,2,3], Sonia Saïd[4], and Vincent Bansaye[1]
1 Ecole Polytechnique, Centre de mathématiques appliquées (CMAP), Palaiseau, 91128, France
2 REsearch LAboratory for Interdisciplinary Studies (RELAIS), Paris, 75012, France
3 Institut national du sport, de l'expertise et de la performance (INSEP), Paris, 75012, France
4 Office Français de la Biodiversité, Direction Recherche et Appui Scientifique, Unité Ongulés Sauvages-Unité Flore et Végétation, Birieux, 01330, France

## Supplementary Table S2

Results of the statistics showing model performance when using only one parameter instead of the three.

| Deer# | Stat | Configuration | mean($e_1$) | std($e_1$) | median($e_1$) | interquartile($e_1$) |
|---|---|---|---|---|---|---|
| 1 | Distribution of turning angles | RW | 0.2910 | 0.0053 | 0.2909 | 0.0072 |
| 1 | Distribution of turning angles | $p_F$ | 0.2912 | 0.0052 | 0.2910 | 0.0070 |
| 1 | Distribution of turning angles | $p_s$ | 0.2914 | 0.0068 | 0.2912 | 0.0093 |
| 1 | Distribution of turning angles | $p_I$ | 0.2910 | 0.0053 | 0.2912 | 0.0071 |
| 1 | Distribution of turning angles | $p_I, p_s, p_F$ | 0.2896 | 0.0068 | 0.2898 | 0.0093 |
| 1 | Home range (Kernel estimate) | RW | 1.42E+08 | 1.67E+07 | 1.42E+08 | 2.15E+07 |
| 1 | Home range (Kernel estimate) | $p_F$ | 1.43E+08 | 1.65E+07 | 1.43E+08 | 2.14E+07 |
| 1 | Home range (Kernel estimate) | $p_s$ | 9.40E+07 | 9.65E+06 | 9.44E+07 | 1.30E+07 |
| 1 | Home range (Kernel estimate) | $p_I$ | 1.42E+08 | 1.74E+07 | 1.41E+08 | 2.20E+07 |
| 1 | Home range (Kernel estimate) | $p_I, p_s, p_F$ | 9.29E+07 | 9.85E+06 | 9.34E+07 | 1.24E+07 |
| 1 | Dilation | RW | 2.08E+06 | 1.28E+06 | 2.00E+06 | 2.08E+06 |
| 1 | Dilation | $p_F$ | 2.14E+06 | 1.28E+06 | 2.14E+06 | 2.11E+06 |
| 1 | Dilation | $p_s$ | 1.97E+06 | 1.21E+06 | 1.94E+06 | 1.93E+06 |
| 1 | Dilation | $p_I$ | 2.09E+06 | 1.24E+06 | 2.08E+06 | 1.97E+06 |
| 1 | Dilation | $p_I, p_s, p_F$ | 2.01E+06 | 1.21E+06 | 1.96E+06 | 1.92E+06 |
| 1 | Immobile transects | RW | 10178.0 | 132.8 | 10189.0 | 155.0 |
| 1 | Immobile transects | $p_F$ | 10174.0 | 130.1 | 10190.0 | 165.5 |
| 1 | Immobile transects | $p_s$ | 10023.0 | 142.6 | 10041.0 | 171.0 |
| 1 | Immobile transects | $p_I$ | 10175.0 | 140.2 | 10190.0 | 152.5 |
| 1 | Immobile transects | $p_I, p_s, p_F$ | 10029.0 | 137.3 | 10051.0 | 160.5 |
| 1 | Mobile transects (linear) | RW | 4916.7 | 424.0 | 4956.5 | 605.5 |
| 1 | Mobile transects (linear) | $p_F$ | 4923.6 | 412.8 | 4957.5 | 555.5 |
| 1 | Mobile transects (linear) | $p_s$ | 4922.4 | 423.8 | 4961.5 | 561.0 |
| 1 | Mobile transects (linear) | $p_I$ | 4898.4 | 438.1 | 4948.5 | 601.0 |
| 1 | Mobile transects (linear) | $p_I, p_s, p_F$ | 4876.2 | 432.4 | 4909.0 | 567.0 |
| 1 | Mobile transects (rotating) | RW | 9994.3 | 594.0 | 10169.0 | 737.5 |
| 1 | Mobile transects (rotating) | $p_F$ | 9948.4 | 632.3 | 10138.0 | 757.0 |
| 1 | Mobile transects (rotating) | $p_s$ | 9876.4 | 690.7 | 10063.0 | 833.5 |
| 1 | Mobile transects (rotating) | $p_I$ | 9946.3 | 642.3 | 10128.0 | 735.0 |
| 1 | Mobile transects (rotating) | $p_I, p_s, p_F$ | 9796.4 | 727.2 | 10009.0 | 939.5 |
| 2 | Distribution of turning angles | RW | 0.2436 | 0.0057 | 0.2437 | 0.0074 |
| 2 | Distribution of turning angles | $p_F$ | 0.2437 | 0.0053 | 0.2436 | 0.0074 |
| 2 | Distribution of turning angles | $p_s$ | 0.2441 | 0.0064 | 0.2441 | 0.0091 |
| 2 | Distribution of turning angles | $p_I$ | 0.2436 | 0.0056 | 0.2437 | 0.0072 |
| 2 | Distribution of turning angles | $p_I, p_s, p_F$ | 0.2468 | 0.0065 | 0.2467 | 0.0089 |
| 2 | Home range (Kernel estimate) | RW | 1.58E+08 | 2.02E+07 | 1.58E+08 | 2.37E+07 |
| 2 | Home range (Kernel estimate) | $p_F$ | 1.58E+08 | 1.99E+07 | 1.57E+08 | 2.44E+07 |
| 2 | Home range (Kernel estimate) | $p_s$ | 1.17E+08 | 1.33E+07 | 1.18E+08 | 1.64E+07 |
| 2 | Home range (Kernel estimate) | $p_I$ | 1.58E+08 | 1.90E+07 | 1.57E+08 | 2.34E+07 |
| 2 | Home range (Kernel estimate) | $p_I, p_s, p_F$ | 1.05E+08 | 1.68E+07 | 1.04E+08 | 2.19E+07 |
| 2 | Dilation | RW | 4.00E+06 | 1.45E+06 | 4.00E+06 | 2.32E+06 |
| 2 | Dilation | $p_F$ | 4.02E+06 | 1.40E+06 | 4.10E+06 | 2.13E+06 |
| 2 | Dilation | $p_s$ | 3.80E+06 | 1.42E+06 | 3.84E+06 | 2.20E+06 |
| 2 | Dilation | $p_I$ | 3.91E+06 | 1.44E+06 | 3.94E+06 | 2.26E+06 |
| 2 | Dilation | $p_I, p_s, p_F$ | 3.92E+06 | 1.18E+06 | 3.83E+06 | 1.77E+06 |
| 2 | Immobile transects | RW | 10783.0 | 135.7 | 10804.0 | 159.0 |
| 2 | Immobile transects | $p_F$ | 10784.0 | 134.6 | 10804.0 | 158.5 |
| 2 | Immobile transects | $p_s$ | 10705.0 | 148.7 | 10733.0 | 186.0 |
| 2 | Immobile transects | $p_I$ | 10786.0 | 131.4 | 10800.0 | 161.0 |
| 2 | Immobile transects | $p_I, p_s, p_F$ | 10617.0 | 219.8 | 10658.0 | 259.0 |
| 2 | Mobile transects (linear) | RW | 3955.0 | 297.3 | 3986.5 | 398.5 |
| 2 | Mobile transects (linear) | $p_F$ | 3961.4 | 294.0 | 3997.0 | 379.5 |
| 2 | Mobile transects (linear) | $p_s$ | 3973.9 | 280.9 | 3994.0 | 387.5 |
| 2 | Mobile transects (linear) | $p_I$ | 3969.3 | 282.9 | 3988.5 | 367.0 |
| 2 | Mobile transects (linear) | $p_I, p_s, p_F$ | 3277.0 | 445.7 | 3287.5 | 627.5 |
| 2 | Mobile transects (rotating) | RW | 5911.9 | 495.9 | 6059.5 | 610.5 |
| 2 | Mobile transects (rotating) | $p_F$ | 5899.6 | 481.5 | 6020.5 | 608.5 |
| 2 | Mobile transects (rotating) | $p_s$ | 5904.6 | 468.4 | 6017.0 | 598.0 |
| 2 | Mobile transects (rotating) | $p_I$ | 5934.5 | 463.1 | 6063.0 | 614.5 |
| 2 | Mobile transects (rotating) | $p_I, p_s, p_F$ | 4608.8 | 830.0 | 4618.0 | 1162.0 |
| 3 | Distribution of turning angles | RW | 0.2539 | 0.0061 | 0.2540 | 0.0083 |
| 3 | Distribution of turning angles | $p_F$ | 0.2537 | 0.0061 | 0.2535 | 0.0077 |
| 3 | Distribution of turning angles | $p_s$ | 0.2546 | 0.0074 | 0.2545 | 0.0098 |
| 3 | Distribution of turning angles | $p_I$ | 0.2542 | 0.0061 | 0.2540 | 0.0086 |
| 3 | Distribution of turning angles | $p_I, p_s, p_F$ | 0.2502 | 0.0070 | 0.2501 | 0.0095 |
| 3 | Home range (Kernel estimate) | RW | 1.53E+08 | 1.89E+07 | 1.52E+08 | 2.10E+07 |
| 3 | Home range (Kernel estimate) | $p_F$ | 1.52E+08 | 1.88E+07 | 1.51E+08 | 2.19E+07 |
| 3 | Home range (Kernel estimate) | $p_s$ | 1.05E+08 | 1.07E+07 | 1.05E+08 | 1.41E+07 |
| 3 | Home range (Kernel estimate) | $p_I$ | 1.53E+08 | 1.98E+07 | 1.51E+08 | 2.12E+07 |
| 3 | Home range (Kernel estimate) | $p_I, p_s, p_F$ | 1.01E+08 | 1.15E+07 | 1.02E+08 | 1.57E+07 |
| 3 | Dilation | RW | 3.29E+06 | 1.41E+06 | 3.35E+06 | 2.20E+06 |
| 3 | Dilation | $p_F$ | 3.23E+06 | 1.41E+06 | 3.15E+06 | 2.19E+06 |
| 3 | Dilation | $p_s$ | 3.27E+06 | 1.34E+06 | 3.39E+06 | 2.04E+06 |
| 3 | Dilation | $p_I$ | 3.41E+06 | 1.41E+06 | 3.45E+06 | 2.19E+06 |
| 3 | Dilation | $p_I, p_s, p_F$ | 3.31E+06 | 1.35E+06 | 3.29E+06 | 2.05E+06 |
| 3 | Immobile transects | RW | 8219.5 | 124.0 | 8238.5 | 142.0 |
| 3 | Immobile transects | $p_F$ | 8221.1 | 117.0 | 8232.5 | 146.0 |
| 3 | Immobile transects | $p_s$ | 8140.2 | 133.7 | 8163.5 | 166.0 |
| 3 | Immobile transects | $p_I$ | 8223.7 | 113.5 | 8238.5 | 140.5 |
| 3 | Immobile transects | $p_I, p_s, p_F$ | 8124.4 | 146.2 | 8148.5 | 165.5 |
| 3 | Mobile transects (linear) | RW | 3805.0 | 297.3 | 3844.0 | 391.0 |
| 3 | Mobile transects (linear) | $p_F$ | 3791.9 | 295.8 | 3814.0 | 398.0 |
| 3 | Mobile transects (linear) | $p_s$ | 3810.2 | 284.6 | 3839.5 | 377.5 |
| 3 | Mobile transects (linear) | $p_I$ | 3785.0 | 288.0 | 3826.0 | 392.5 |
| 3 | Mobile transects (linear) | $p_I, p_s, p_F$ | 3611.9 | 358.0 | 3665.5 | 491.0 |
| 3 | Mobile transects (rotating) | RW | 8521.5 | 559.2 | 8686.0 | 648.0 |
| 3 | Mobile transects (rotating) | $p_F$ | 8538.0 | 511.2 | 8655.0 | 686.0 |
| 3 | Mobile transects (rotating) | $p_s$ | 8511.4 | 540.2 | 8657.5 | 636.0 |
| 3 | Mobile transects (rotating) | $p_I$ | 8518.4 | 553.5 | 8687.0 | 627.0 |
| 3 | Mobile transects (rotating) | $p_I, p_s, p_F$ | 8095.0 | 815.8 | 8315.0 | 1057.5 |
| 4 | Distribution of turning angles | RW | 0.2440 | 0.0062 | 0.2438 | 0.0084 |
| 4 | Distribution of turning angles | $p_F$ | 0.2442 | 0.0061 | 0.2443 | 0.0078 |
| 4 | Distribution of turning angles | $p_s$ | 0.2444 | 0.0073 | 0.2444 | 0.0105 |
| 4 | Distribution of turning angles | $p_I$ | 0.2438 | 0.0060 | 0.2436 | 0.0082 |
| 4 | Distribution of turning angles | $p_I, p_s, p_F$ | 0.2406 | 0.0067 | 0.2407 | 0.0093 |
| 4 | Home range (Kernel estimate) | RW | 1.38E+08 | 1.99E+07 | 1.37E+08 | 2.20E+07 |
| 4 | Home range (Kernel estimate) | $p_F$ | 1.39E+08 | 2.07E+07 | 1.36E+08 | 2.12E+07 |
| 4 | Home range (Kernel estimate) | $p_s$ | 9.74E+07 | 1.09E+07 | 9.76E+07 | 1.48E+07 |
| 4 | Home range (Kernel estimate) | $p_I$ | 1.37E+08 | 1.94E+07 | 1.35E+08 | 2.17E+07 |
| 4 | Home range (Kernel estimate) | $p_I, p_s, p_F$ | 9.18E+07 | 1.25E+07 | 9.19E+07 | 1.67E+07 |
| 4 | Dilation | RW | 2.64E+06 | 1.37E+06 | 2.64E+06 | 2.17E+06 |
| 4 | Dilation | $p_F$ | 2.59E+06 | 1.35E+06 | 2.59E+06 | 2.00E+06 |
| 4 | Dilation | $p_s$ | 2.50E+06 | 1.31E+06 | 2.55E+06 | 2.04E+06 |
| 4 | Dilation | $p_I$ | 2.70E+06 | 1.39E+06 | 2.72E+06 | 2.11E+06 |
| 4 | Dilation | $p_I, p_s, p_F$ | 2.63E+06 | 1.22E+06 | 2.61E+06 | 1.86E+06 |
| 4 | Immobile transects | RW | 5369.7 | 313.3 | 5402.0 | 419.0 |
| 4 | Immobile transects | $p_F$ | 5370.1 | 297.2 | 5405.0 | 385.0 |
| 4 | Immobile transects | $p_s$ | 5523.5 | 313.2 | 5547.5 | 431.0 |
| 4 | Immobile transects | $p_I$ | 5363.3 | 308.1 | 5396.0 | 394.0 |
| 4 | Immobile transects | $p_I, p_s, p_F$ | 5340.8 | 366.2 | 5387.5 | 506.0 |
| 4 | Mobile transects (linear) | RW | 2999.6 | 326.7 | 3033.5 | 429.5 |
| 4 | Mobile transects (linear) | $p_F$ | 2998.7 | 317.6 | 3033.0 | 419.0 |
| 4 | Mobile transects (linear) | $p_s$ | 3004.7 | 306.8 | 3020.5 | 420.5 |
| 4 | Mobile transects (linear) | $p_I$ | 2973.5 | 314.5 | 3010.0 | 421.5 |
| 4 | Mobile transects (linear) | $p_I, p_s, p_F$ | 2720.3 | 393.4 | 2761.0 | 532.0 |
| 4 | Mobile transects (rotating) | RW | 7056.5 | 584.9 | 7234.0 | 683.5 |
| 4 | Mobile transects (rotating) | $p_F$ | 7077.2 | 590.2 | 7257.0 | 689.0 |
| 4 | Mobile transects (rotating) | $p_s$ | 7021.6 | 578.3 | 7176.0 | 728.5 |
| 4 | Mobile transects (rotating) | $p_I$ | 7032.4 | 569.2 | 7199.0 | 710.0 |
| 4 | Mobile transects (rotating) | $p_I, p_s, p_F$ | 6385.6 | 918.6 | 6543.0 | 1346.5 |
| 5 | Distribution of turning angles | RW | 0.2440 | 0.0067 | 0.2440 | 0.0093 |
| 5 | Distribution of turning angles | $p_F$ | 0.2444 | 0.0065 | 0.2445 | 0.0088 |
| 5 | Distribution of turning angles | $p_s$ | 0.2442 | 0.0077 | 0.2442 | 0.0104 |
| 5 | Distribution of turning angles | $p_I$ | 0.2445 | 0.0064 | 0.2443 | 0.0089 |
| 5 | Distribution of turning angles | $p_I, p_s, p_F$ | 0.2458 | 0.0075 | 0.2456 | 0.0104 |
| 5 | Home range (Kernel estimate) | RW | 1.43E+08 | 1.89E+07 | 1.42E+08 | 2.20E+07 |
| 5 | Home range (Kernel estimate) | $p_F$ | 1.44E+08 | 1.92E+07 | 1.43E+08 | 1.97E+07 |
| 5 | Home range (Kernel estimate) | $p_s$ | 9.76E+07 | 9.84E+06 | 9.79E+07 | 1.28E+07 |
| 5 | Home range (Kernel estimate) | $p_I$ | 1.43E+08 | 1.77E+07 | 1.42E+08 | 2.04E+07 |
| 5 | Home range (Kernel estimate) | $p_I, p_s, p_F$ | 8.35E+07 | 1.74E+07 | 8.03E+07 | 2.23E+07 |
| 5 | Dilation | RW | 4.08E+06 | 1.38E+06 | 4.17E+06 | 2.09E+06 |
| 5 | Dilation | $p_F$ | 4.07E+06 | 1.40E+06 | 4.07E+06 | 2.10E+06 |
| 5 | Dilation | $p_s$ | 3.85E+06 | 1.35E+06 | 3.86E+06 | 2.03E+06 |
| 5 | Dilation | $p_I$ | 4.16E+06 | 1.40E+06 | 4.17E+06 | 2.15E+06 |
| 5 | Dilation | $p_I, p_s, p_F$ | 3.91E+06 | 1.11E+06 | 3.90E+06 | 1.57E+06 |
| 5 | Immobile transects | RW | 7113.1 | 174.9 | 7133.0 | 217.5 |
| 5 | Immobile transects | $p_F$ | 7118.3 | 172.7 | 7141.5 | 211.0 |
| 5 | Immobile transects | $p_s$ | 7110.6 | 181.6 | 7141.0 | 229.5 |
| 5 | Immobile transects | $p_I$ | 7117.0 | 178.7 | 7146.5 | 217.0 |
| 5 | Immobile transects | $p_I, p_s, p_F$ | 6566.2 | 454.3 | 6623.5 | 590.0 |
| 5 | Mobile transects (linear) | RW | 2973.3 | 284.4 | 3005.0 | 381.0 |
| 5 | Mobile transects (linear) | $p_F$ | 2978.3 | 290.1 | 3000.5 | 390.5 |
| 5 | Mobile transects (linear) | $p_s$ | 2992.5 | 275.5 | 3020.0 | 346.5 |
| 5 | Mobile transects (linear) | $p_I$ | 2988.9 | 281.2 | 3013.0 | 387.5 |
| 5 | Mobile transects (linear) | $p_I, p_s, p_F$ | 1907.4 | 443.2 | 1905.0 | 634.0 |
| 5 | Mobile transects (rotating) | RW | 6987.0 | 496.1 | 7126.0 | 563.5 |
| 5 | Mobile transects (rotating) | $p_F$ | 6963.1 | 505.5 | 7100.5 | 628.0 |
| 5 | Mobile transects (rotating) | $p_s$ | 6964.0 | 500.3 | 7105.0 | 611.0 |
| 5 | Mobile transects (rotating) | $p_I$ | 7015.8 | 448.2 | 7142.0 | 523.5 |
| 5 | Mobile transects (rotating) | $p_I, p_s, p_F$ | 4427.2 | 1074.3 | 4417.5 | 1546.0 |
